# BTLA suppress acute rejection via regulating TCR downstream signals and cytokines production in kidney transplantation and prolonged allografts survival

**DOI:** 10.1038/s41598-019-48520-7

**Published:** 2019-08-21

**Authors:** Jiayi Zhang, Hengcheng Zhang, Zijie Wang, Haiwei Yang, Hao Chen, Hong Cheng, Jiajun Zhou, Ming Zheng, Ruoyun Tan, Min Gu

**Affiliations:** Department of Urology, First Affiliated Hospital of Nanjing Medical University, Nanjing, 210029 China

**Keywords:** Allotransplantation, Experimental models of disease, Acute inflammation

## Abstract

Acute rejection is a major risk for renal transplant failure. During this adverse process, activated T cells are considered the main effectors. Recently, B and T lymphocyte attenuator (BTLA), a member of the CD28 family receptor, was reported to be a novel inhibitory regulator of T cell activation in heart and pancreatic allograft rejection. Due to the similarity of acute rejection pathways among different organs, we hypothesized that BTLA might play a role in acute rejection of kidney transplant. In renal transplant patients, we observed that BTLA expression was significantly decreased in peripheral CD3+ T lymphocytes of biopsy-proven acute rejection (BPAR) recipients compared with control patients with stable transplanted kidney functions. Remarkably, overexpression of BTLA in the rat model was found to significantly inhibit the process of acute rejection, regulate the postoperative immune status, and prolong allograft survival. BTLA overexpression significantly suppressed IL-2 and IFN-γ production and increased IL-4 and IL-10 production both *in vivo* and *in vitro*. Moreover, vital factors in T-cell signaling pathways, including mitogen-associated protein kinases (MAPK), nuclear factor-kappa B (NF-κB) and nuclear factor of activated T cells (NFAT), were also significantly repressed by BTLA overexpression. Therefore, BTLA can suppress acute rejection and regulate allogeneic responses of kidney transplant by regulating TCR downstream signals and inflammatory cytokines production to improve allografts outcomes.

## Introduction

Kidney transplantation, which is the optimal therapeutic intervention for end-stage renal disease (ESRD), could provide a high quality of life and longer survival when compared with maintenance dialysis^1,2^. Short-term graft survival and recipient survival after the operation have been substantially increased by potent immunosuppressants targeting T lymphocytes^3,4^. However, late kidney allograft survival is still strongly affected by various immunological and nonimmunological factors^5–8^. Among these factors, acute cellular rejection (ACR), characterized by mononuclear cellular infiltrates in the allograft, has been recognized as a powerful predictor and major risk of graft outcomes including chronic rejection^9–11^. In a study of a consecutive series of 319 cases receiving cadaveric renal transplants over a 7-year period, 31% had a single early acute rejection episode^12^. However, the potential mechanisms of acute rejection following renal transplantation remain unclear and require further investigation.

T cells are major coordinators and effectors of transplant rejection^13^, and T cell–mediated rejection (TCMR) with T-cell activation and differentiation is the major category of ACR^14^. Co-stimulatory and co-inhibitory receptors are recognized to regulate the balance of activation of T cells, indicating that T-cell receptor signaling without co-stimulation may progress to anergy and apoptosis. The co-signaling receptor B and T lymphocyte attenuator (BTLA, or CD272), with structural similarities to programmed cell death protein 1 (PD-1) and cytotoxic T-lymphocyte antigen-4 (CTLA-4), is the most recently identified CD28 family member^15–17^. BTLA expression is induced in most mature lymphocytes including activated CD4+ and CD8+ T cells, B cells, macrophages, dendritic cells (DCs) and NK cells^18^. It has been reported that BTLA-deficient T cells exhibit increased proliferation upon antigen stimulation presented by DCs, suggesting that BTLA might exert an inhibitory signal on T cells^19^. However, the specific impact of BTLA on organ transplantation has been rarely investigated. As previously reported, targeting of BTLA in alloreactive CD4+ and CD8+ T cells prolongs graft survival in mouse cardiac graft models^20^. Additionally, little is known with respect to the function of BTLA in acute rejection after renal transplant.

Mitogen-associated protein kinases (MAPK) and nuclear factor of activated T cells (NFAT) have been reported to play pivotal role in regulating T cell development, activation, differentiation, and the induction of T cell tolerance^21–23^. Moreover, nuclear factor-kappa B (NF-κB) is a critical transcription factor for the development of inflammation^24^. A microarray analysis of allogeneic kidney transplantation using a rat model indicated an induction of NFATc1 and NF-κB-related pathways^25^. Furthermore, it was confirmed that ligation of BTLA in the Jurkat T cell line resulted in a remarkable downregulation of NF-κB, NFAT and AP-1 activity^26^. Therefore, we hypothesized that BTLA had an inhibitory role in acute rejection following kidney transplantation via suppression of T cell activation signaling pathways.

As previously described in our preliminary study, the BTLA/HVEM pathway is involved in the pathogenesis of acute rejection following kidney transplantation, as well as the induction of transplant tolerance^27^. In the present study, we continue to study the role of BTLA in a larger cohort of renal allograft acute rejection patients. We also apply rat renal allograft acute rejection models as well as an *ex vivo* mixed lymphocyte reaction (MLR) to investigate the effect of BTLA on acute rejection of renal transplant. By overexpression of BTLA, we observed significant alleviation of the acute rejection with prolonged renal allograft survival in allografted rats. BTLA acted through the suppression T cell activation (especially CD4+ T cells), as evidenced by the reduced activation of MAPK and NF-κB signaling and lower Th1-related interleukin secretion (IL-2 and IFN-γ). These findings highlight BTLA as a promising therapeutic target for acute rejection relief and immunologic tolerance induction in kidney transplantation patients.

## Results

### Decreased expression of BTLA in recipients with acute rejection

Twenty patients with acute renal transplant rejection (BPAR group) were included in this study. For comparison, the same number of patients with stable renal allograft function (Stable group) and healthy individuals (Control group) were also recruited. The main characteristics of these patients and healthy donors are presented in Table 1. Among the features listed in the table, serum creatinine (SCr) and serum blood urea nitrogen (BUN) levels of the BPAR group were significantly higher than those in the Stable group or Control group (SCr: 318.12 ± 116.2 μmol/L vs 90.56 ± 4.89 μmol/L/77.95 ± 3.31 μmol/L, *P* < 0.001; BUN: 17.51 ± 1.82 mmol/L vs 4.98 ± 1.01 mmol/L/4.43 ± 0.39 μmol/L *P* < 0.001). Additionally, no significant difference in other clinical characteristics was observed among the three groups.

**Table 1:** Demographic and basic characteristics of enrolled cases in this study.

	BPAR(n = 20)	Stable(n = 20)	Control(n = 20)	*P* value
Age (years; mean ± SD)	44.26 ± 4.01	45.19 ± 3.85	41.25 ± 3.35	NS
Gender (male/female)	15/5	16/4	10/10	NS
Transplant duration (years; range)	0.61 (0.1–3.6)	0.9 (0.8–3.7)	/	NS
HLA mismatch (mean ± SD)	4.60 ± 0.25	4.93 ± 0.32	/	NS
Primary/secondary kidney transplant	20/0	20/0	/	NS
**Donor source**				NS
Living-related	2	2	/	
Cadaveric	18	18	/	
**Immunosuppressive regimen**				NS
Prednisone + MMF + CsA	12	10	/	
Prednisone + MMF + Tac	8	10	/	
**Biochemical parameters**				
Serum creatinine (μmol/L; mean ± SD)	318.12 ± 116.2	90.56 ± 4.89	77.95 ± 3.31	< 0.001
BUN (mmol/L; mean ± SD)	17.51 ± 1.82	4.98 ± 1.01	4.43 ± 0.39	< 0.001

Abbreviations: BPAR: biopsy-proven acute rejection; HLA: human leukocyte antigen; MMF: mycophenolate mofetil; CsA: cyclosporine A; Tac: tacrolimus; BUN: blood urea nitrogen; SD: standard deviation; NS: no significance.

**Figure 1:**
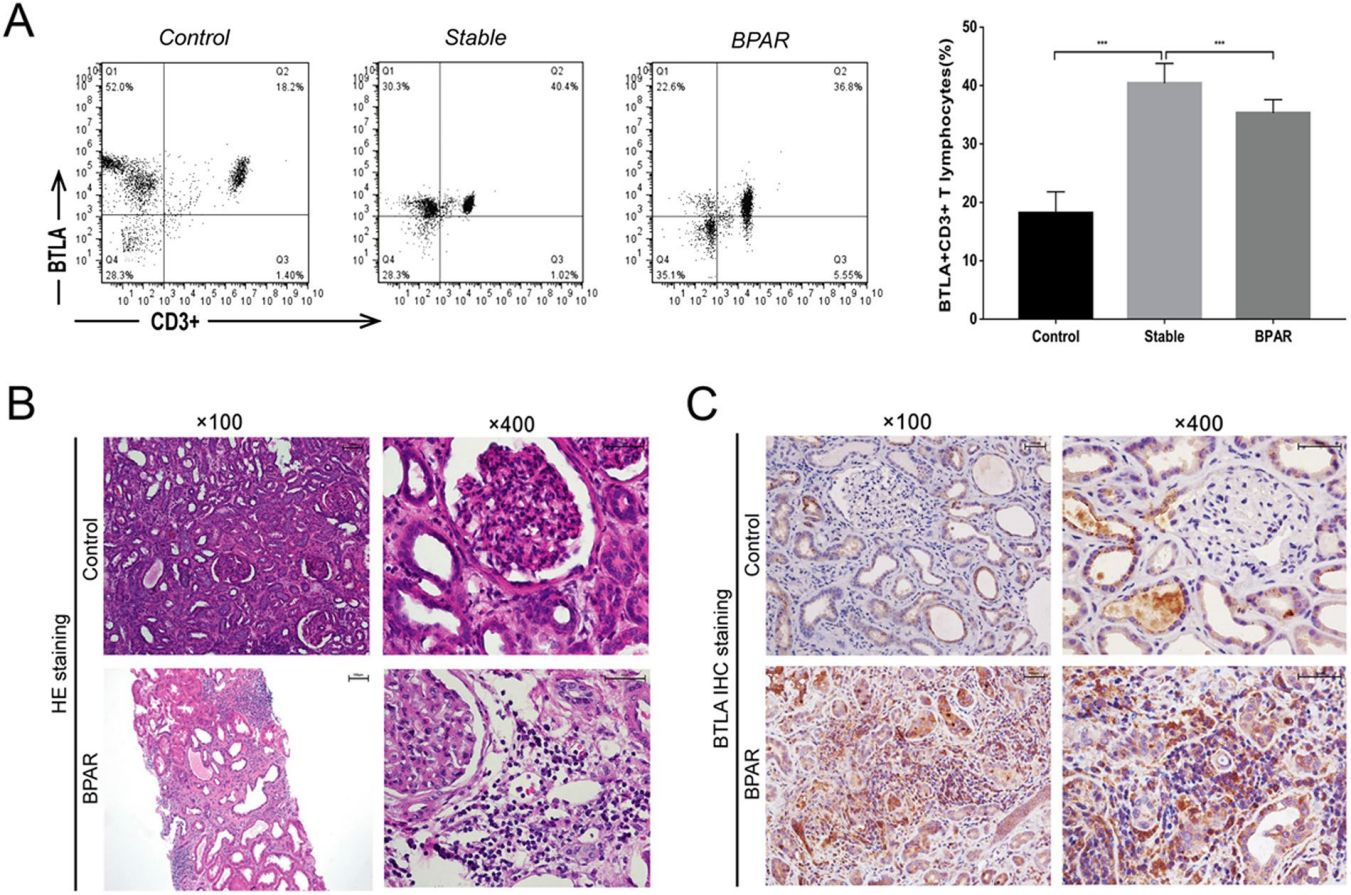
BTLA expression in human recipients among the Control, BPAR and Stable groups. (**A**) Flow cytometry was used to compare BLTA expression on the surface of CD3+ T cells in peripheral blood mononuclear cells (PBMCs) obtained from the BPAR and Stable groups, and the percentage of BTLA+ cells among CD3+ T cells. Data are means ± SD. **P < 0.01. (**B**) HE staining showing the histological features of allograft tissue among the Control and BPAR groups following transplantation (magnifications: 100X and 400X). (**C**) BTLA protein expression by immunohistochemical staining (IHC) in the BPAR and control groups.

With the peripheral blood from the three different groups, flow cytometry analysis showed that the expression of BTLA on CD3+ T lymphocytes in recipients with BPAR was statistically lower than that with stable allograft function (*P* < 0.01) (Fig. 1A). Moreover, BTLA expression was markedly higher in recipients after transplantation than in healthy volunteers (Fig. 1A). In BPAR patient biopsy tissue, we observed significant mononuclear cell infiltration and edema in the interstitial tissue from the allograft after HE staining (Fig. 1B). No renal biopsy could be obtained from the Stable group or healthy donor; instead, we used normal tissues from radical nephrectomy of renal cancer patients as normal control (Fig. 1B,C). The IHC staining results showed an overexpression of BTLA in allograft samples from the BPAR group compared with the control group (Fig. 1C). These results confirmed our previous report and encouraged us to further investigate the roles of BTLA in renal acute rejection.

### Establishment of the renal allograft acute rejection model

**Figure 2:**
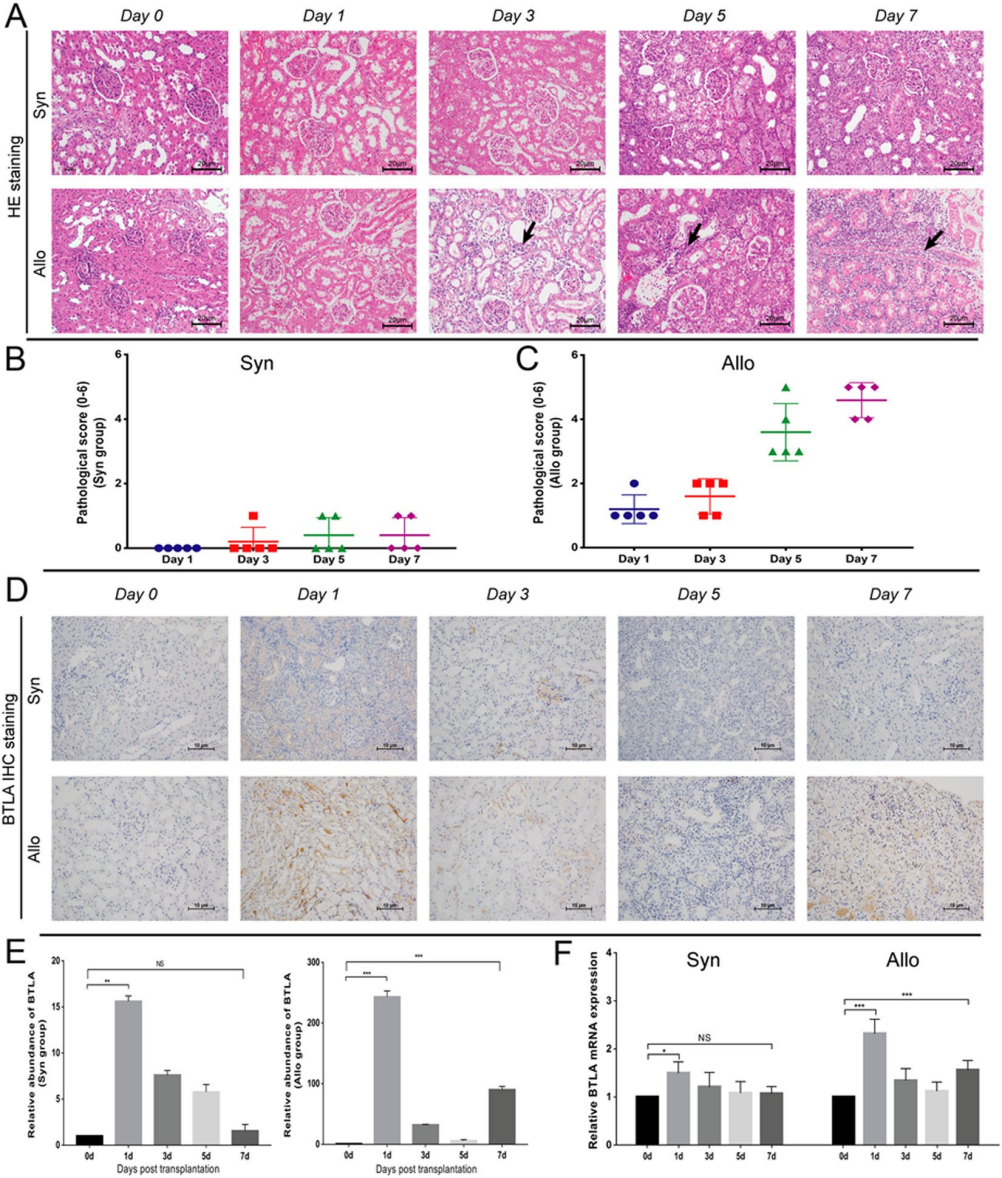
Establishment of the rat renal allograft acute rejection model and expression of BTLA in grafts. (**A**) HE staining of the histological features of renal tissue between the Syn and Allo groups at Day 0, 1, 3, 5, and 7 following transplantation (magnification: 200x). Arrow: monocyte infiltration. (**B**,**C**) Semi-quantitative assessment of graft tissues from the Syn and Allo groups based on the Banff 2017 classification at Day 0, 1, 3, 5, and 7 after kidney transplantation (n = 5 for each time point). (**D**) Postoperative BTLA protein expression of kidney grafts by IHC in the Syn and Allo groups (magnification: 200x). (**E**) Integrated optical density (IOD) value used to express the relative quantity of BTLA protein. (**F**) Postoperative mRNA expression of BTLA in kidney grafts by qRT-PCR in the Syn and Allo groups. The control was a non-treated SD rat (Day 0 among each groups). Data are expressed as the means ± SD. NS, not significant, *P < 0.05, **P < 0.01 and ***P < 0.001 versus the no-treated rat.

To further study the regulatory role of BTLA in the acute rejection process, we established a rat renal transplant model of acute rejection confirmed by pathology. As demonstrated by HE staining (Fig. 2A), mild kidney acute injury, such as ischemia reperfusion injury and renal tubule edema, resulting from the operation were observed in the Syn group, without graft acute rejection from Day 1 (D1) to Day 7 (D7) following surgery. By contrast, renal HE staining of the Allo group exhibited mild acute injury in the renal interstitium on D1 after graft transplant. Further renal tubular injury and monocyte infiltration were observed from Day 3 (D3) to Day 5 (D5), which then progressed to severe intimal arteritis on D7. The pathological score based on the Banff 2017 classification clearly revealed the progress of acute rejection from D3 to D7 after transplantation (Fig. 2B,C).

### BTLA expression increased at the early stage of acute rejection

BTLA expression in renal allograft tissues were detected by IHC, and normal SD kidney tissue was considered as a D0 control (Fig. 2D,E). In the recipients of syngeneic (Syn) group, the expression of BTLA slightly increased on D1 after transplantation compared with a normal SD rat, and it subsequently decreased to a low level. In the allogeneic (Allo) group, BTLA expression was significantly increased at D1 with a greater than 200-fold change compared with D0 after the operation, gradually decreased at D3 and D5, and then slightly increased on D7. In addition, BTLA expression in the Allo group showed a markedly higher level at all time points after transplantation compared with the Syn group (*P* < 0.05). Additionally, qRT-PCR demonstrated a similar trend in the alteration of BTLA expression as shown by IHC, but the fold changes were not as extensive as the protein levels observed in both the Syn and Allo groups (Fig. 2F). Therefore, we concluded that there was a significant increase in BTLA expression during the pathogenesis of acute rejection, especially at the early stage, suggesting a potential relationship between BTLA and acute rejection.

### Overexpression of BTLA significantly attenuated graft acute rejection and improved grafts survival

**Figure 3:**
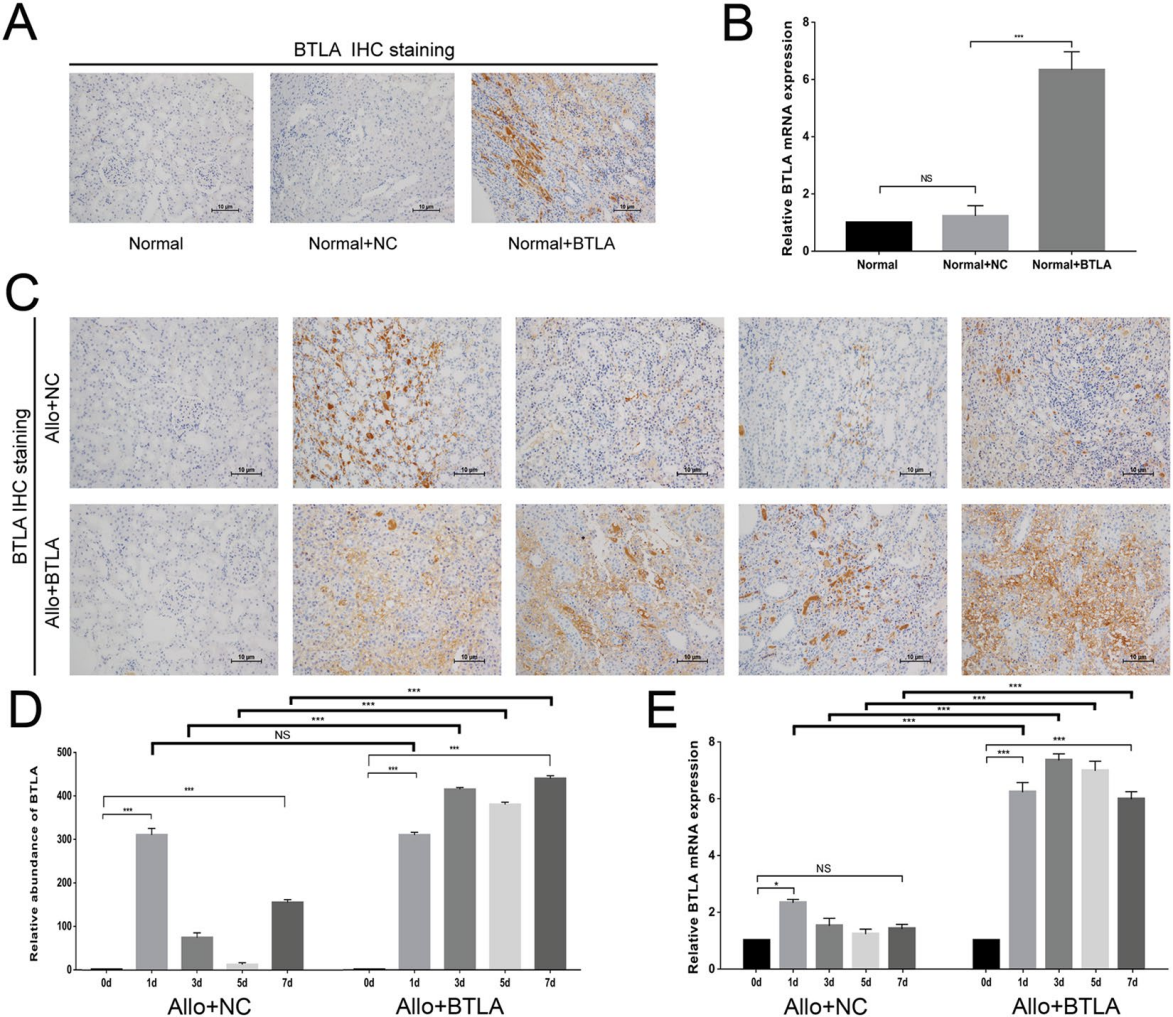
Expression of BTLA in rat allografts in the Allo + NC and Allo + BTLA groups. (**A**) IHC staining showed significantly upregulated BTLA expression in the kidney tissue of the SD rat by BTLA-overexpression adenovirus (Normal + BTLA) compared with the negative control vector (Normal + NC) and normal SD rat (Normal). (**B**) qRT-PCR showed upregulated mRNA expression of BTLA induced by the BTLA-overexpression adenovirus. (**C**) Postoperative BTLA expression of the kidney graft by IHC staining between the Allo + NC and Allo + BTLA groups (magnification: 200×). (**D**) Integrated optical density (IOD) value for quantifying BTLA protein. (**E**) mRNA expression of BTLA in the kidney graft by qRT-PCR among the Allo + NC and Allo + BTLA groups. Data are expressed as the means ± SD. NS, not significant, *P < 0.05, **P < 0.01 and ***P < 0.001.

To clarify the effect of BTLA in acute rejection after kidney transplantation, we overexpressed BTLA expression in the renal allograft acute rejection model as described in Methods. A preliminary experiment showed that BTLA expression of kidney tissues was significantly induced by BTLA-overexpressed adenovirus in SD rats compared with normal SD rats, while almost no BTLA expression was found in the NC group or normal group (Fig. 3A,B).

**Figure 4:**
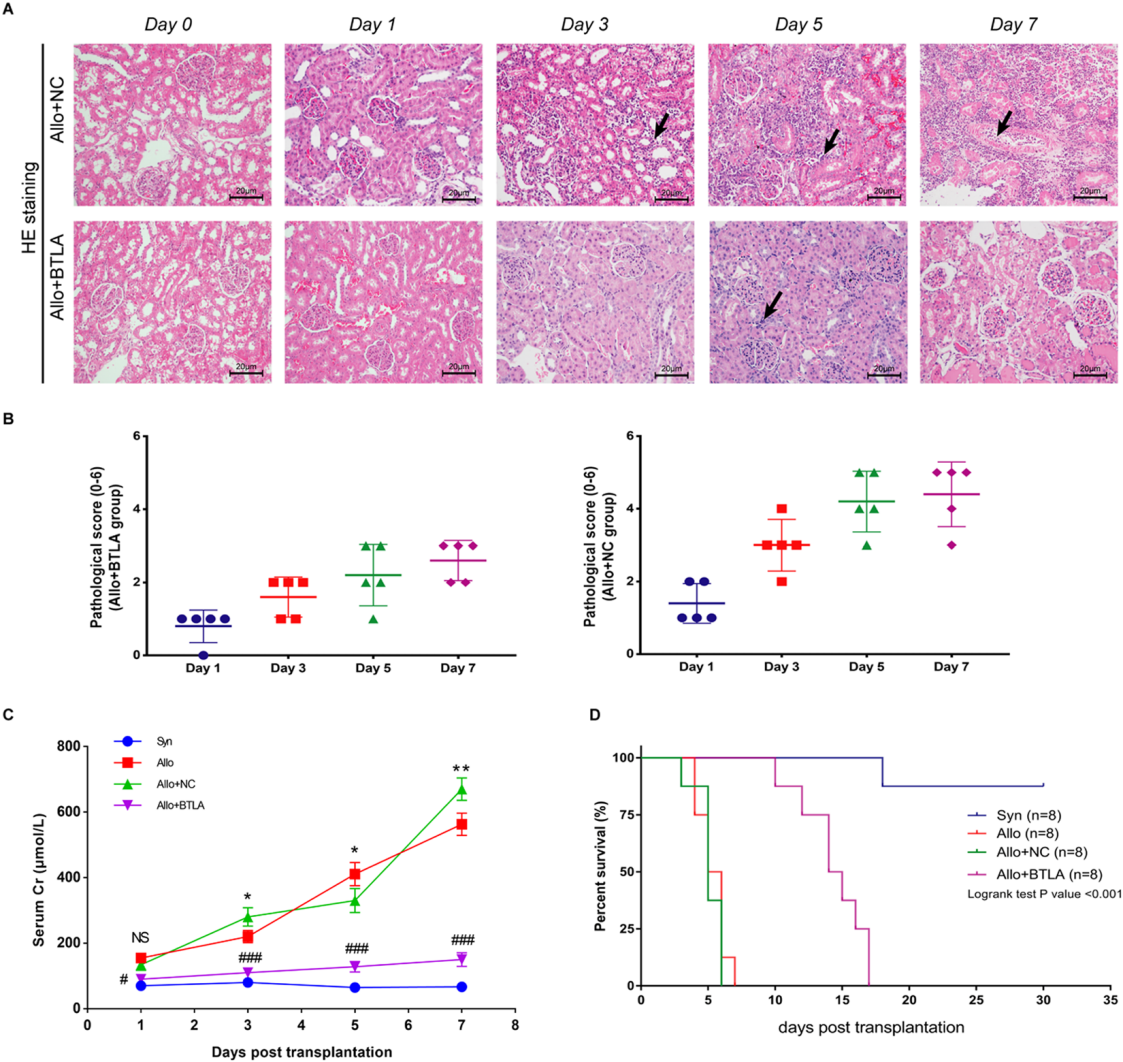
Overexpression of BTLA significantly attenuates graft acute rejection and improves graft survival. (**A**) HE staining of the histological features of renal tissue between the Allo + NC and Allo + BTLA groups at Day 0, 1, 3, 5, and 7 following transplantation (magnification: 200×). Arrow: monocyte infiltration. (**B**) Semi-quantitative assessment of graft tissues based on the Banff classification at Day 1, 3, 5, and 7 after kidney transplantation (n = 5 for each time point). (**C**) Serum creatinine (SCr) among the four groups on Day 0, 1, 3, 5, and 7 after surgery. Data are presented as the means ± SD. For the Allo group vs the Allo + NC group: NS, not significant, *P < 0.05, **P < 0.01 and ***P < 0.001. For the Allo + NC group vs the Allo + BTLA group: ^#^*P* < 0.05 and ^###^*P* < 0.001. (**D**) Rat graft survival among the four groups after renal transplantation.

The expression of BTLA in the Allo + BTLA group was significantly increased versus the Allo + NC group since D3 posttransplant (*P* < 0.05), and no difference was found between the Allo and Allo + NC groups (Fig. 3C–E). Allograft tissues of Allo + BTLA group showed significantly reduced renal injury with monocyte infiltration compared with the Allo + NC group, suggesting a considerable attenuation of the acute rejection process by BTLA overexpression (Fig. 4A). Additionally, the pathological score based on the Banff classification demonstrated a reduction of graft acute rejection after renal transplant in the Allo + BTLA group compared with the Allo + NC group (Fig. 4B,C).

SCr assay of the serum samples from D1, D3, D5, and D7 after surgery showed that recipients of Allo + BTLA group showed a higher SCr level than those in the Syn group but a significantly lower concentration when compared with the Allo + NC group (*P* < 0.05). Besides, no significant difference existed between Allo group and Allo + NC group (P = 0.9673) (Fig. 4D). In addition, recipients in the Allo + BTLA group had significantly better graft survival than those in the Allo + NC group (HR = 0.25, 95% CI: 0.07–0.85, *P* < 0.001), indicating that overexpression of BTLA contributed to the improvement of acute rejection episodes and renal allograft outcomes (Fig. 4E).

### BTLA overexpression significantly contributed to immunosuppressive status *in vivo*

**Figure 5:**
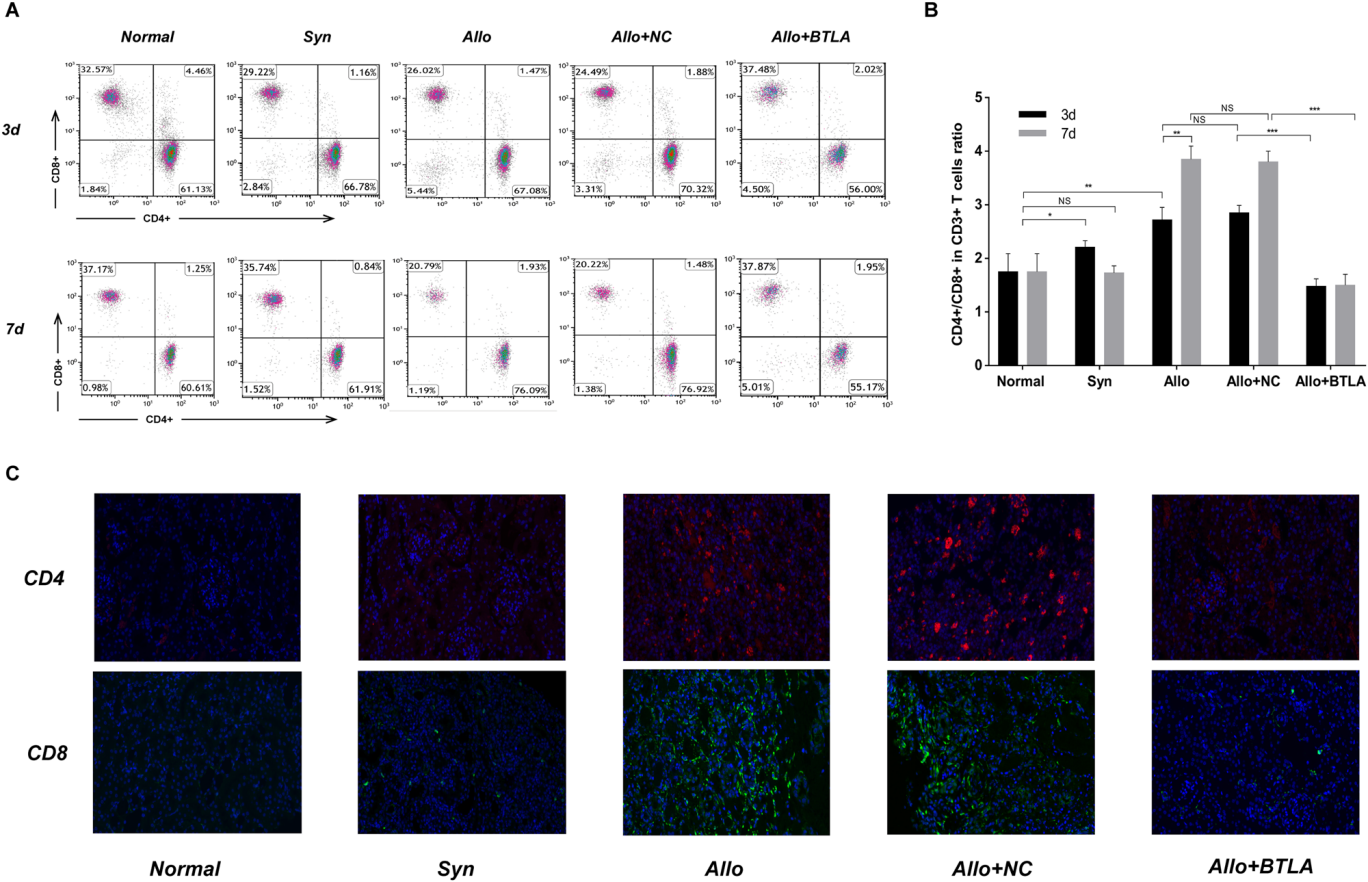
Postoperative immune status of rat recipients. (**A**) Flow cytometry analysis of CD4+ and CD8+ T cells in CD3+ T cells of peripheral blood cells of SD recipients at D3 and D7 following kidney transplantation (n = 5 in each group at each time point). (**B**) The ratio of postoperative CD4+ and CD8+ T cells in CD3+ T cells of SD recipients at D3 and D7 following kidney transplantation. Data are presented as the means ± SD. NS, not significant, *P < 0.05, **P < 0.01 and ***P < 0.001. (**C**) Immunofluorescence assay of graft tissue sections stained with CD4 and CD8 antibodies among the different groups at D7 following kidney transplantation. Magnification: 40× for CD4 and CD8.

To investigate the influence of BTLA on the posttransplant systematic immune status, we performed flow cytometry analysis of PBMCs from rat recipients (Fig. 5A,B). Recipients in the Syn group showed minor CD4+ activation on the 3^rd^ day after surgery and then a similar CD4+/CD8 + ratio (CD4+ versus CD8 + ) as normal SD rats on the 7^th^ day. Significant immune hyperfunction with massive activation of CD4+ T cells was observed among recipients in the Allo group, which resulted in acute rejection of the graft. We found that there was no difference of acute rejection between D1 and D3, or between D5 and D7 in Allo group. Besdies, there was no difference between the Allo and Allo + NC groups. In Allo + BTLA group, we found a significantly lower postoperative CD4+/CD8+ T cell ratio both on D3 and D7 when compared with the Allo + NC group, indicating an immunosuppressive status caused by BTLA overexpression *in vivo*. Based on the immunofluorescence assay, the graft tissues showed higher levels of CD4+- and CD8 + -T cell interstitial infiltration for the Allo group compared with the normal control and Syn group. In the Allo + BTLA group, a reduced presence of CD4+- and CD8 + -T cell infiltration was induced by BTLA overexpression when compared with the Allo + NC groups (Fig. 5C).

### BTLA overexpression inhibited the proliferation of T cells in MLR culture

**Figure 6:**
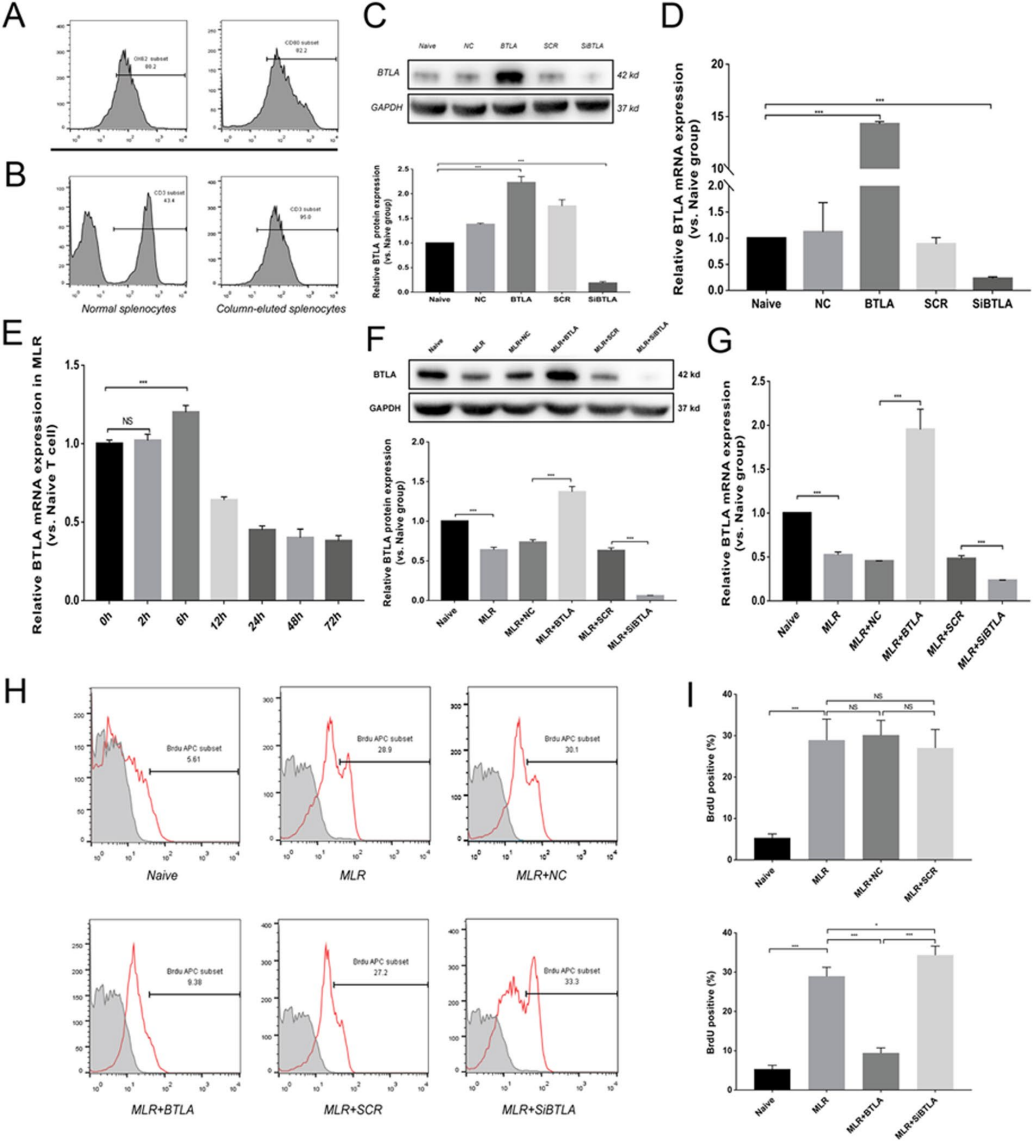
Effect of BTLA in the mixed lymphocyte reaction. (**A**) Flow cytometry analyses of OX62 and CD80 on primary mature DCs from the Wistar rat. (**B**) Flow cytometry analysis of CD3 on splenic lymphocytes from the naive SD rat and purified splenic lymphocytes from the SD rat. (**C**) Relative BTLA protein expression in splenic lymphocytes among different transfected groups by western blot analysis. (**D**) Relative BTLA mRNA expression in splenic lymphocytes from different transfected groups by qRT-PCR. (**E**) Relative BTLA mRNA expression in the MLR group at 0 h, 2 h, 6 h, 12 h, 24 h, 48 h and 72 h by qRT-PCR. (**F**) Protein level of BTLA among different transfected MLR groups by western blot analysis. (**G**) Relative BTLA mRNA expression among different transfected MLR groups by qRT-PCR. (**H**,**I**) BrdU-positive cell percentage among different transfected MLR groups by flow cytometry analysis. The results are presented as the means ± SD of three independent experiments. NS, not significant, *P < 0.05, **P < 0.01 and ***P < 0.001.

Primary mature DCs of Wistar rats were determined by flow cytometry analysis with the specific surface antigens OX62 of 80.2% positive and CD80 of 82.2% positive (Fig. 6A). Total naive T cells were selected from splenic lymphocytes of SD rats with 95.0% purity by flow cytometry (Fig. 6B). Before the MLR assays, BTLA expression on pan T cells was first confirmed by western blot analysis and qRT-PCR in the different treated groups (Fig. 6C,D).

In the MLR assay, we first monitored the alteration of BTLA mRNA expression levels at different time points and observed a slight but significant increase in BTLA mRNA expression at 6 h followed by a clear drop after 12 h in the MLR group vs Naive group (Fig. 6E). The BTLA expression level among the different treated groups was determined at 72 h after MLR by both western blot analysis and qRT-PCR (Fig. 6F,G). Compared with the Naive group, the expression of BTLA in the MLR group was significantly downregulated, demonstrating a decrease of approximately 40%. Moreover, no difference in BTLA expression level was found between the MLR + NC and MLR + SCR groups. BTLA expression in the MLR + BTLA group revealed a significant increase when compared with the MLR + NC group. The MLR + SiBTLA group showed an obvious decrease in BTLA expression compared with the MLR + SCR group. Based on BrdU staining, a markedly increased percentage of positive cells was observed in four groups (29.62 ± 0.95% for MLR, 30.50 ± 0.97% for MLR + NC, 27.84 ± 1.06% for MLR + SCR, and 33.05 ± 1.64% for MLR + SiBTLA) compared with the Naive group (5.95 ± 0.22%). In the MLR + BTLA group, BrdU-positive cells were significantly decreased after 72 h of MLR (10.44 ± 0.86%), suggesting that BTLA overexpression could effectively inhibit T cell proliferation of MLR *in vitro* (Fig. 6H,I).

### BTLA reduced the release of IL-2 and IFN-γ and induced the release of IL-4 and IL-10 *in vivo* and *in vitro*

**Figure 7:**
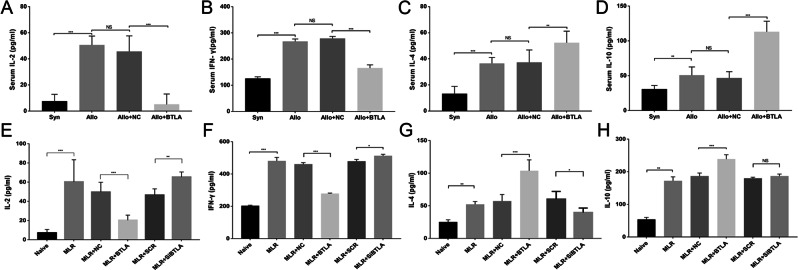
Secretion of cytokines in the acute renal transplant rejection rat model and in the MLR. (**A**–**D**) Rat serum cytokines, including (**A**) IL-2, (**B**) IFN-γ, (**C**) IL-4, and (among the different groups at D7 follow) IL-10, were measured on D7 in the different transfected groups by ELISA. (**E**–**H**) The cytokines (**A**) IL-2, (**B**) IFN-γ, (**C**) IL-4, and (**D**) IL-10 at 72 h of MLR were assayed by ELISA in the different transfected MLR groups.

To understand the systematic immune response status in the rat acute renal transplant rejection model, we measured serum levels of several inflammatory cytokines on D7 among recipients by ELISA. Compared with the Syn group, the Allo and Allo + NC groups showed greater release of IL-2, IFN-γ, IL-4 and IL-10. However, significantly decreased release of IL-2 and IFN-γ was observed in the Allo + BTLA group compared with the Allo + NC group (Fig. 7A,B). Moreover, the Allo + BTLA group exhibited notably higher release of IL-4 and IL-10 than the Allo + NC group (Fig. 7C,D).

By contrast, secretion of cytokines in the supernatant of the MLR culture was also determined by ELISA at 72 h. Consistent with the results using the rat models, the secretion of IL-2 and IFN-γ was significantly downregulated in the MLR + BTLA group compared with the MLR + NC group (Fig. 7E,F), together with upregulated secretion of IL-4 and IL-10 when BTLA was overexpressed (Fig. 7G,H). By contrast, the secretion of IL-2 and IFN-γ only rose slightly in the MLR + SiBTLA group compared with the MLR + SCR group. Interestingly, we did not find a significant difference in IL-10 release in the MLR + SiBTLA group compared with the MLR + SCR group.

### Overexpression of BTLA inhibited specific molecules of TCR signaling pathways

**Figure 8:**
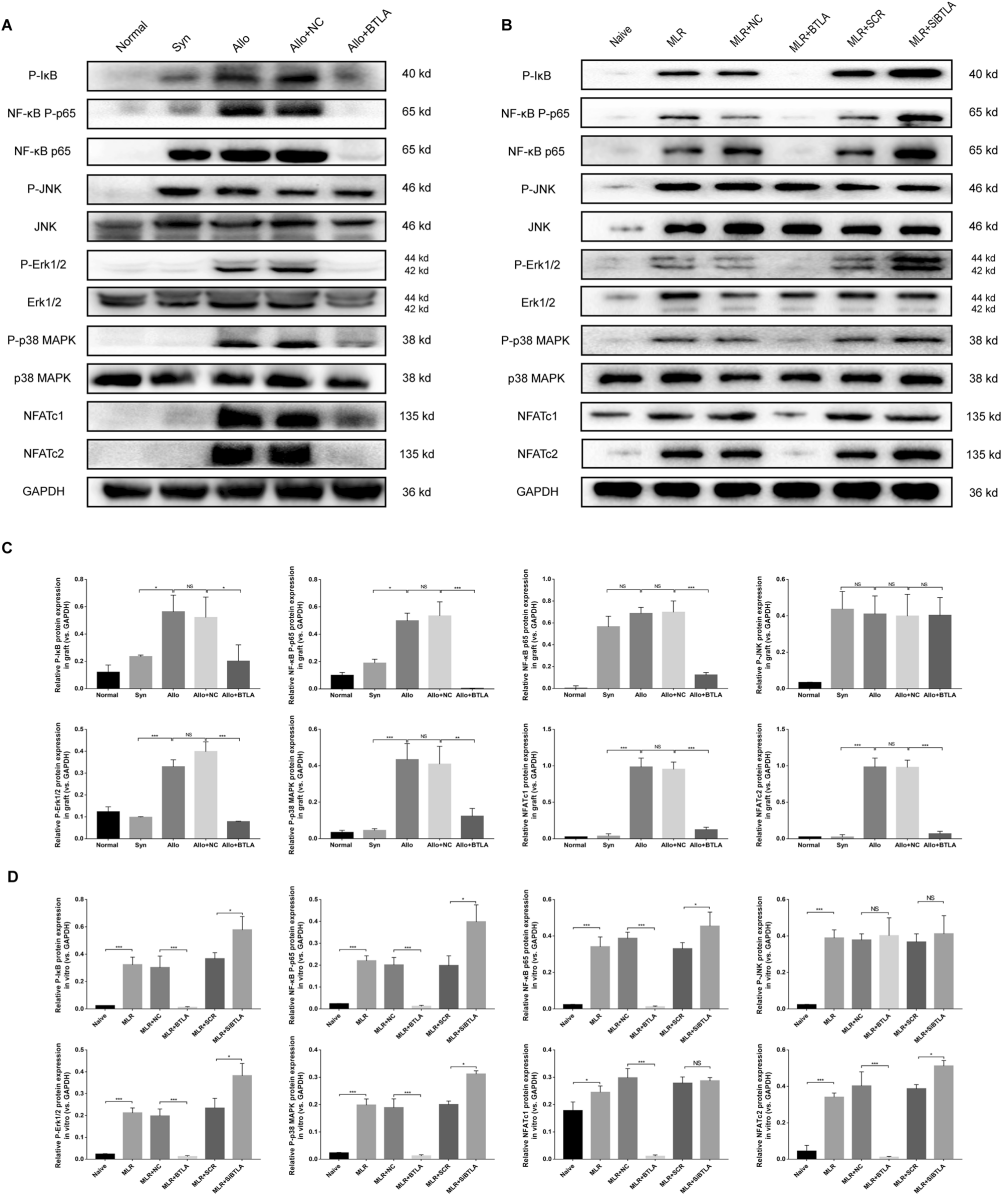
Expression of downstream factors during TCR signaling activation in the acute renal transplant rejection rat model and in MLR. Protein expression levels of P-IκB, NF-κB P-P65, NF-κB P65, P-Erk1/2, Erk1/2, JNK, P-JNK, P-p38 MAPK, p38 MAPK, NFATc1 and NFATc2 among the groups *in vivo* (**A**,**C**) and *in vitro* (**B**,**D**). The grouping of gels/blots cropped from different parts of the same gel or from different gels. The results are representative of three independent experiments.

Downstream of TCR activation signaling, ERK1/2, NFAT and NF-κB are the major molecules involved^28^. *In vivo*, we extracted total protein of allografts and observed slightly elevated expression of P-IκB, NF-κB P-p65, NF-κB p65, P-JNK and JNK in the Syn group compared with the normal group (Fig. 8A,C). In the Allo group, P-IκB, NF-κB P-p65, P-ERK1/2, P-p38 MAPK, and NFATc1/c2 expression levels were significantly increased compared with the Syn group, suggesting that the NF-κB, MAPK and NFAT signaling pathways were primarily activated in the progression of acute rejection. In the Allo + BTLA group, we found that in the context of BTLA overexpression, the expression of P-IκB, NF-κB P-p65, NF-κB p65, P-ERK 1/2, P-p38 MAPK, and NFATc1/c2 was significantly downregulated compared with the Allo + NC group. No difference was observed in the JNK pathway between the Allo + BTLA and Allo + NC groups.

*In vitro*, we also detected activation of the NF-κB, MAPK and NFAT signaling pathways after MLR (Fig. 8B,D). Similar to the *in vivo* results, overexpression of BTLA significantly downregulated the expression of P-IκB, NF-κB P-p65, NF-κB p65, P-ERK 1/2, P-p38 MAPK, and NFATc1/c2 in the MLR + BTLA compared with the MLR + NC group. In addition, we did not find any significant difference in P-JNK and JNK expression among the MLR groups.

## Discussion

Acute rejection remains a major challenge in kidney transplantation, shortening allograft survival, and there is an urgent need for predictive biomarkers and an effective immunosuppressive theory. Recent reports have engaged the improvement of allograft survival via co-inhibitory signals, especially in the BTLA pathway^19,20,29^. In this study, we collected clinical samples and developed a rat renal allograft model with MLR culture to investigate the potential effect of the coinhibitory factor BTLA in acute rejection and tolerance of kidney transplantation.

As a T lymphocyte inhibitory receptor, BTLA was found to be one of the most highly induced genes selectively expressed in anergic CD4+ T cells, but it was not highly expressed on naturally occurring CD25+ T regulatory cells, unlike CTLA-4 and PD-1^18^. In the renal allograft acute rejection model, we observed consistently increased CD4+ T cell counts with a higher CD4+/CD8+ T cell ratio in Allo compared with Syn recipients. Moreover, the acute rejection process at D1 following the operation seemed to be mild but deteriorated after D3 to D7. Interestingly, the BTLA expression level in the Allo group was dynamically altered, peaking at D1 following transplant and then decreasing from D3 to D5 but rising slightly on D7. This phenomenon might imply that as a negative co-stimulatory receptor, BTLA expression is upregulated during the early stage of acute rejection and then rapidly reduced due to excessive consumption. Similar to these results, decreased BTLA expression in peripheral blood of BPAR recipients also demonstrated a process of BTLA consumption. Thus, we overexpressed BTLA in the renal transplant rat model to investigate the potential underlying mechanism. As expected, acute rejection was significantly attenuated by BTLA overexpression in recipients, along with reduced pathological injury and a lower Banff score, as compared to the Allo group. Stable BTLA overexpression also contributed to superior renal function with lower SCr levels in the recipients, along with better graft survival. BTLA overexpression led to the immunosuppression of recipients, with a decrease in CD4+ T cell counts and the CD4+/CD8+ T cell ratio. The expression of CD4+ and CD8+ in recipient grafts was also downregulated in the context of BTLA overexpression. Remarkably, we found that BTLA overexpression led to a significant reduction of T cell proliferation in MLR culture. These results provide evidence for our hypothesis that BTLA overexpression might have a strong influence on reducing T cell activation and proliferation in renal acute rejection.

We also explored the possible role of BTLA in affecting the balance of inflammatory cytokine production, which was involved in T cell activation and proliferation. The helper T-cells (Th), Th1, are known to contribute highly to graft rejection^30^, and IL-2 and IFN-γ are cytokines produced by the Th1 cells of activated alloreactive CD4+ T cells^16^. Gene products of IL-2 and its receptor are considered essential for T-cell proliferation^13^. Moreover, IL-4 and IL-10 are commonly secreted by Th2 cells^31^. As previously reported, most acute rejection specimens exhibit high IFN-γ expression and low IL-4 expression^30,32,33^. In the present study, a significant decrease in IL-2 and IFN-γ along with a significant increase in IL-4 and IL-10 by BTLA overexpression were detected in the renal acute rejection model, as well as in the BTLA-overexpression MLR culture. With anti-inflammatory and suppressive effects on alloimmune responses, Th2 cell-produced IL-4 and IL-10 may also shift the immune response from the Th1 pathway to Th2 responses, favoring Ig production. This result could imply that Th2-related cytokines attenuate the rejection process and protect allografts^34^. In addition, IFN-γ has been reported to have antiproliferative effects on emerging Th2 cells because only Th2 cells express both chains of the IFN-γ receptors^35^. Thus, inducing the secretion of IL-4 and IL-10 in the context of BTLA overexpression has also been considered as feedback for reducing the secretion of IFN-γ^36^.

In addition, we focused on specific molecules of signaling pathways downstream of the TCR, which included MAPKs (p38 MAPK, JNK and ERK1/2), NF-κB, and NFAT^28,37,38^. Our results showed that the phosphorylation of these molecules was induced by allograft acute rejection. Moreover, a significant inhibitory effect of BTLA overexpression on the phosphorylation of IκB, NF-κB p65, ERK 1/2, and p38 MAPK, and on the expression of NF-κB p65 and NFATc1/2, was observed *in vivo* and *in vitro*. Moreover, knockdown of BTLA *in vitro* was found to induce the activation of these signaling molecules. The unchanged expression of P-JNK indicated that the JNK signaling pathway might not be involved in the BTLA-mediated reduction of acute rejection.

Taken together, these findings strongly suggest a vital inhibitory role of BTLA in the costimulatory pathway by repressing the activation of TCR downstream pathways and transcription factors that regulate the production of cytokines (e.g., IL-2, IFN-γ, IL-4 and IL-10), subsequently leading to a reduction of T cell proliferation and shift in T cell differentiation, which could further influence cytokine production. Therefore, the suppressive effect of BTLA was verified in renal allograft acute rejection by reducing T cell activation and proliferation, resulting in the prolongation of allograft survival. This is the first study to examine the potential role of BTLA in the rat renal allograft acute rejection model. There are some advantages of our research. First, orthotopic graft transplant by the standard microsurgery technique using end-to-end anastomosis was performed in all rat models, which is similar to the environment of the human renal transplant. Second, BTLA expression among the groups of transplant models was similar to our previous result in human recipients^27^.

In conclusion, our findings suggest that BTLA played an inhibitory role in acute rejection by regulating allogeneic responses of renal graft through TCR downstream signaling pathways and cytokines production. Hence, BTLA may be considered a promising target for immunologic tolerance and long-term graft survival in kidney transplantation.

## Materials and Methods

### Ethics statement

The study protocol was conducted in accordance with the ethical standards of the Declarations of Helsinki and Istanbul, and it was approved by the local ethics committee of the First Affiliated Hospital of Nanjing Medical University (2016-SR-029). No organs/tissues were procured from (executed) prisoners. Donor kidneys were allocated directly by the Organ Procurement Organization (OPO), the First Affiliated Hospital of Nanjing Medical University. All donors or next of kin freely provided their written informed consent for participation.

Patients and samples A total of 20 allograft segments and peripheral blood samples were obtained from biopsy-proven acute rejection (BPAR) recipients who had undergone surgery for kidney transplantation between December 1, 2011 to December 1, 2017 at our center. The inclusion and exclusive criteria have been previously described^27^. Moreover, we collected blood samples of 20 renal transplant recipients in our center with stable allograft function, defined as a serum creatinine (SCr) level less than 120 µmol/L for at least 3 months following kidney transplantation. Blood samples were also gathered from 20 healthy volunteers who were considered the control group. Additionally, 10 normal kidney samples, which were more than 5 cm away from the tumor tissues, were obtained from patients who had undergone radical nephrectomy. For the cases of renal recipients with BPAR, blood samples were obtained immediately when the clinical symptoms occurred and before the allograft biopsy and administration of high-dose chemotherapy with methylprednisolone.

### Experimental animals

Male Wistar and Sprague Dawley (SD) rats weighing 200–250 g were purchased from Charles River Laboratory (Beijing, China). All rats were housed in standard rat cages and maintained under a 12-h/12-h light/dark cycle with water and a normal diet in Nanjing Medical University Animal Center. All experimental procedures were approved by the Animal Care and Use Committee of Nanjing Medical University (Ethical Approval Number: IACUC1601140–1), and all experiments were performed in accordance with relevant guidelines and regulations.

### Rat kidney transplantation

Paired rats were divided into four surgical groups. For the syngeneic (Syn) group, surgery was conducted between the SD rat donor and SD rat recipient. For the allogeneic (Allo) group, the Wistar rat was the donor, and the SD rat was the recipient. For the Allo + BTLA group, the SD recipient was intravenously injected with the BTLA-overexpression adenovirus (Genechem Shanghai, China) 48 h before renal transplant. For the Allo + NC group, the SD rat was pre-treated with a corresponding negative control (NC) vector (CMV-MCS-3FLAG-SV40-EGFP) (Genechem Shanghai, China) 48 h before surgery.

The renal transplants were performed by two experienced microsurgeons (JY Zhang and HC Zhang) under a surgical microscope at 10X magnification (Supplementary Fig. 1). The syngeneic or allogeneic donor was anesthetized with 2% isoflurane, and the left kidney was mobilized and perfused as previously described before being placed in University of Wisconsin solution at 4 °C for 15 min^39^. The SD recipient was anesthetized, and left nephrectomy was conducted. The operation was performed first with arterial anastomosis between the recipient renal artery and the donor renal artery by the end-to-end technique using 10-0 Prolene sutures (Johnson, NJ, USA). The recipient renal vein and donor renal vein were then anastomosed using the same standard technique. Finally, the ureter was anastomosed to the recipient bladder, and a right nephrectomy was subsequently performed. A recipient was considered eligible for further research if the graft became bright red ≤ 20 s after release of the microvascular clamps.

At every time point (Day 1, 3, 5, and 7) following surgery, five rats from each group were sacrificed for collection of kidney tissues and peripheral blood. The Day 0 (D0) sample was collected from the normal SD rat. Additionally, the other eight recipients in each group were enrolled in the follow-up of graft survival, and the failure of grafts was defined as anuria. We analyzed the postoperative graft function of rat recipients by measuring the serum creatinine level (SCr) using the Creatinine Kit (Jiancheng BI, Nanjing, China) at each observation time point (D0 to D7).

### Hematoxylin-eosin (HE) staining

The kidney graft tissues from rat recipients were fixed in 10% buffered formalin. The sample tissues were cut into 3-μm-thick sections and stained with HE staining according to standard protocols. Acute rejection was evaluated by two experienced renal pathologists, H Chen and R Tan, in a blinded manner with reference to the Banff 2017 classification^40^. The severity of acute rejection was assessed semi-quantitatively according to a previously reported method^41^, with a score value of 0 as normal, 1 as borderline change, 2 as IA, 3 as IB, 4 as IIA, 5 as IIB, and 6 as III. Antibody-mediated rejection by the Banff classification was not presented in our study, and this grade was not added in the scoring.

### Immunohistochemistry (IHC) and immunofluorescence (IF) assays

Immunohistochemical staining was conducted to investigate the expression of BTLA in kidney graft sections as previously described^42^. The primary BTLA antibody (Abbiotec CA, USA) along with HRP-conjugated secondary antibody was used. Eight images under a high power field (200×) were captured randomly from each specimen in each group using Image Pro Plus 5.0 software (Media Cybernetics MD, USA), with the integrated optical density (IOD) value to express the relative quantity of BTLA. Images were captured using a Nikon Eclipse 50i microscope (Nikon, Japan) along with Imaging Software NIS-Elements (Nikon, Japan).

For immunofluorescence staining, frozen tissue sections of kidney grafts were blocked with goat serum for 30 min at room temperature and then incubated with anti-rat CD4 and anti-rat CD8 primary antibodies (Santa Cruz CA, USA) at 4 °C overnight and with FITC goat anti-rat IgG at 37 °C for 1 h.

### Mixed lymphocyte reaction (MLR)

Splenic T cells of SD rats were purified using a T cell isolation kit (Miltenyi Biotec CA, USA) according to the manufacturer’s protocols as the responder cells, and they were cultured in 12-well plates. Mature DCs, which function as stimulators in the MLR, were separated from the peripheral blood of Wistar rats by the adherent method^20^ and then cultured for 7–9 days in RPMI 1640 medium (Gibco, Grand Island NY, USA) containing 50 ng/ml rat GM-CSF, 10 ng/ml IL-4, and 20 ng/ml TNF-α. Mature DCs were pre-treated with mitomycin C (30 mg/ml, Santa CA, USA) for 20 min before the MLR cultures.

Six groups (Naive, MLR, MLR + NC, MLR + BTLA, MLR + SCR, MLR + SiBTLA) of MLR cultures were established *in vitro*. Naive T cells were pre-treated with BTLA adenovirus (BTLA-overexpress for the MLR + BTLA group and BTLA-knockdown for the MLR + SiBTLA group), and corresponding negative control vectors (BTLA-overexpress control, CMV-MCS-3FLAG-SV40-EGFP for the MLR + NC group and BTLA-knockdown control, hU6-MCS-CMV-EGFP for the MLR + SCR group) for 48 h before MLR according to the manufacturer’s protocol (Genechem Shanghai, China). MLR cultures were maintained in complete medium for 72 h as previously described^43^. Total RNA of isolated T cells in the MLR group was collected at 0 h, 2 h, 6 h, 12 h, 24 h, 48 h, and 72 h for quantitative real-time PCR analysis of BTLA expression.

### Flow cytometry analysis

Analysis of BTLA expression on CD4+ lymphocytes in peripheral blood mononuclear cells (PBMCs) of patients from the BPAR and stable groups was performed as previously described^27^. Peripheral blood cells of rat recipients at D3 and D7 following kidney transplantation were collected and then stained with APC-labeled anti-CD3 (eBioscience CA, USA), FITC-labeled anti-CD4 (eBioscience CA, USA), and PercCP-eFluor710-labeled anti-CD8 (eBioscience CA, USA) antibodies at 4 °C for 45 min for flow cytometry analysis. APC-labeled anti-OX62 (eBioscience CA, USA) along with PE-labeled anti-CD80 (BioLegend CA, USA) were used to determine the purity of the primary DCs, and APC-labeled anti-CD3 was used for T cells. Cell staining was performed according to the manufacturers’ recommendations. T cell proliferation was measured by bromodeoxyuridine (BrdU) incorporation (APC BrdU Flow Kit, BD Pharmingen NJ, USA) according to the manufacturer’s protocols. Cells stained with APC-labeled anti-BrdU mAb (BD Pharmingen NJ, USA) were analyzed by flow cytometry. Flow cytometry analysis was conducted using a Gallios flow cytometer (Beckman Coulter, USA).

### Quantitative reverse transcription-PCR (qRT-PCR) analysis

Total RNA was extracted from graft tissues and MLR cells using RNA extraction kits (Tiangen, China) according to the manufacturer’s protocol. cDNA was synthesized using the PrimeScript RT reagent Kit (Takara, Japan). qRT-PCR was performed with a SYBR Green PCR kit (TaKaRa Biotechnology, Japan) on a StepOnePlus™ Real-Time PCR System (Applied Biosystems, USA). The following primers were used for qRT-PCR:

BTLA, forward: 5′-ATCCCAGATGCTACCAATGC-3′,

reverse: 5′-TTGGGAGTTTGTCCTGGAAC-3′;

GAPDH, forward: 5′-GGCCTTCCGTGTTCCTACC-3′,

reverse: 5′-CGCCTGCTTCACCACCTTC-3′.

Fold changes in mRNA expression were calculated using the 2^−ΔΔCt^ method and normalized based on GADPH with ABI Step One Software version 2.1. Experiments were repeated at least three times.

### Western blot analysis

For protein extraction, cells were lysed in RIPA buffer containing protease inhibitors (Sigma Aldrich, USA). Protein concentration was measured using the BCA Protein Assay (Thermo Fisher Scientific, Inc., USA). Equal amounts of protein samples were separated by 10% SDS-PAGE and blotted onto a polyvinylidene fluoride (PVDF) membrane (Millipore, Billerica MA, USA) for immunoblotting assays. The primary antibodies included anti-rat BTLA (Abbiotec CA, USA), NFATc1 (Santa Cruz CA, USA), NFATc2 (Santa Cruz Biotechnology, CA, USA), P-IκB (CST, Inc., USA), NF-κB P-P65 (CST, Inc., USA), NF-κB P65 (CST, Inc., USA), JNK (CST, Inc., USA), P-JNK (CST, Inc., USA), ERK1/2 (CST, Inc., USA), P-ERK 1/2 (CST, Inc., USA), p38 MAPK (CST, Inc., USA), and P-p38 MAPK (CST, Inc., USA), or GAPDH (CST, Inc., USA) at 4 °C. The chemiluminescent reaction (ECL) was used to reveal the bands, and the band intensity and volume were determined using Image Lab software (Bio-Rad CA, USA).

### Enzyme-linked immunosorbent assay (ELISA)

ELISA was carried out using specific kits (Cusabio Wuhan, China) to detect IL-2, IL-4, IL-10, and IFN-γ concentrations in recipient serum at D7 following renal transplant, as well as in the supernatants of the MLR culture at 72 h. The optical density (OD) value at 450 nm was measured on the microplate reader (Bio-Rad, USA). All procedure was performed strictly according to the manufacturer’s protocols.

### Statistical analysis

Graft survival was estimated using Kaplan-Meier analysis and the log-rank test. The hazard ratio (HR) and corresponding 95% confidence interval (CI) were determined to calculate the survival significance of BTLA overexpression compared with the NC group. Comparisons between groups were subjected to One-way ANOVA. All data were expressed as the mean ± standard deviations (SDs) from three independent experiments. A two-sided p-value < 0.05 was considered statistically significant. Analyses were performed using SPSS 21.0 software (SPSS, Inc., Chicago, IL, USA).

## Supplementary information

supplementary file

## Data Availability

The datasets generated during and/or analyzed during the current study are available from the corresponding author on reasonable request.
