# Author Correction: BTLA suppress acute rejection via regulating TCR downstream signals and cytokines production in kidney transplantation and prolonged allografts survival

**DOI:** 10.1038/s41598-022-24559-x

**Published:** 2022-12-07

**Authors:** Jiayi Zhang, Hengcheng Zhang, Zijie Wang, Haiwei Yang, Hao Chen, Hong Cheng, Jiajun Zhou, Ming Zheng, Ruoyun Tan, Min Gu

**Affiliations:** Department of Urology, First Affiliated Hospital of Nanjing Medical University, Nanjing, 210029 China

Correction to: *Scientific Reports* https://doi.org/10.1038/s41598-019-48520-7, published online 21 August 2019

This Article contains errors in Figure 3.

In Figure 3A, the immunohistochemical staining figure in the Normal+NC group is incorrect.

In addition, Figure 3C was incomplete. The days 0 to 7 are added.

The correct Figure 3 and accompanying legend appears below.

**Figure 3:**
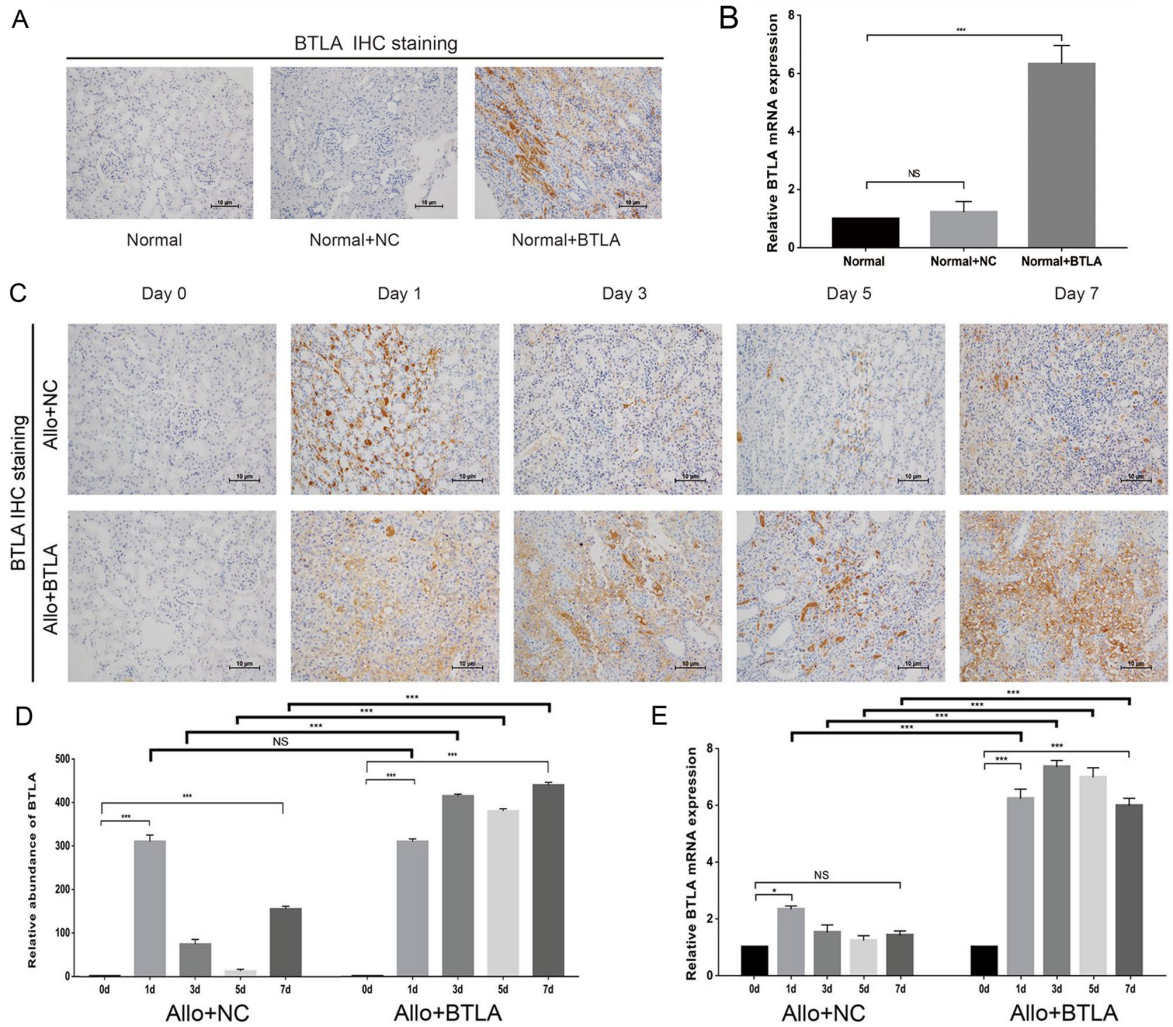
Expression of BTLA in rat allografts in the Allo + NC and Allo + BTLA groups. (**A**) IHC staining showed significantly upregulated BTLA expression in the kidney tissue of the SD rat by BTLA-overexpression adenovirus (Normal + BTLA) compared with the negative control vector (Normal + NC) and normal SD rat (Normal). (**B**) qRT-PCR showed upregulated mRNA expression of BTLA induced by the BTLA-overexpression adenovirus. (**C**) Postoperative BTLA expression of the kidney graft by IHC staining between the Allo + NC and Allo + BTLA groups (magnification: 200×). (**D**) Integrated optical density (IOD) value for quantifying BTLA protein. (**E**) mRNA expression of BTLA in the kidney graft by qRT-PCR among the Allo + NC and Allo + BTLA groups. Data are expressed as the means ± SD. NS, not significant, *P < 0.05, **P < 0.01 and ***P < 0.001.

