# Supplementary material for: BTLA suppress acute rejection via regulating TCR downstream signals and cytokines production in kidney transplantation and prolonged allografts survival
Source: Sci Rep. 2019 Aug 21;9:12154. doi: 10.1038/s41598-019-48520-7 (PMC6704067; doi:10.1038/s41598-019-48520-7)
Supplement: Supplementary file 1 — supplementary file [file 41598_2019_48520_MOESM1_ESM.pdf]

BTLA suppress acute rejection via regulating TCR downstream signals and cytokine production in kidney transplantation and prolong allograft survival

Jiayi Zhang[1]*, Hengcheng Zhang[1]*, Zijie Wang[1]*, Haiwei Yang[1], Hao Chen[1], Hong Cheng[1], Jiajun Zhou[1], Ming Zheng[1], Ruoyun Tan[1], Min Gu[1]△

[1] Department of Urology, First Affiliated Hospital of Nanjing Medical University, Nanjing 210029, China.

* Equal contributors

△Corresponding author: Dr. Min Gu, Ph.D., M.D.,

Department of Urology, the First Affiliated Hospital of Nanjing Medical University, Nanjing 210029, China.

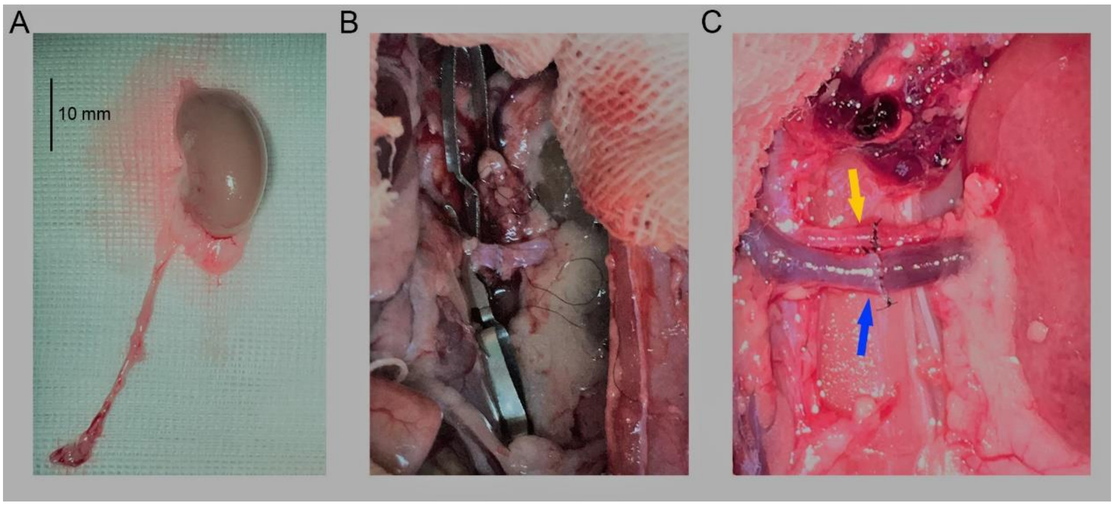

Supplementary Figure 1. Renal transplant in the rat model. (A) Donor left kidney with artery, vein and whole ureter after perfusion, (B) The renal artery and vein were blocked using microvascular clamps before the anastomosis was completed, (C) The blood flow of the graft recovered through the renal artery (yellow arrow) and vein (blue arrow) after release of the microvascular clamps.

Supplementary Information. Full-length gels and blots used for *in vivo* and *in vitro* western blot analyses (see below).

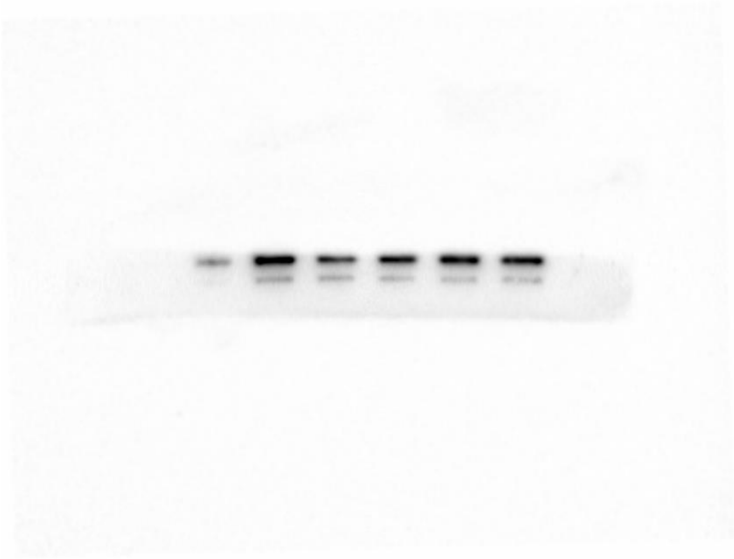

vitro_Erk

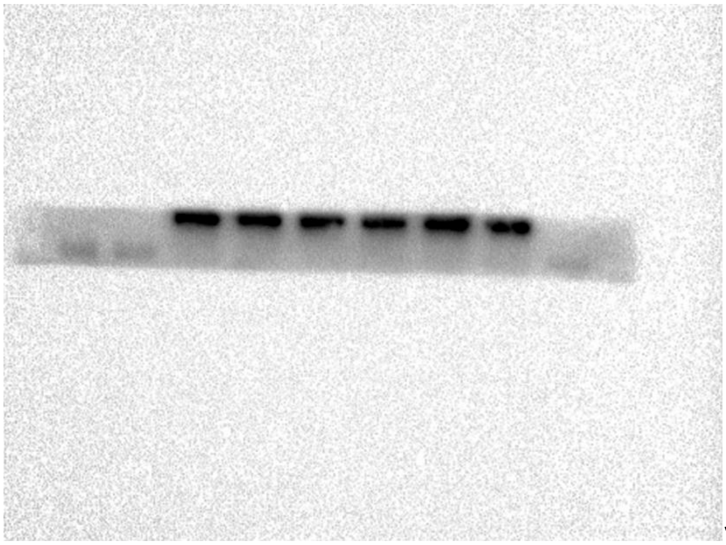

vitro_GAPDH

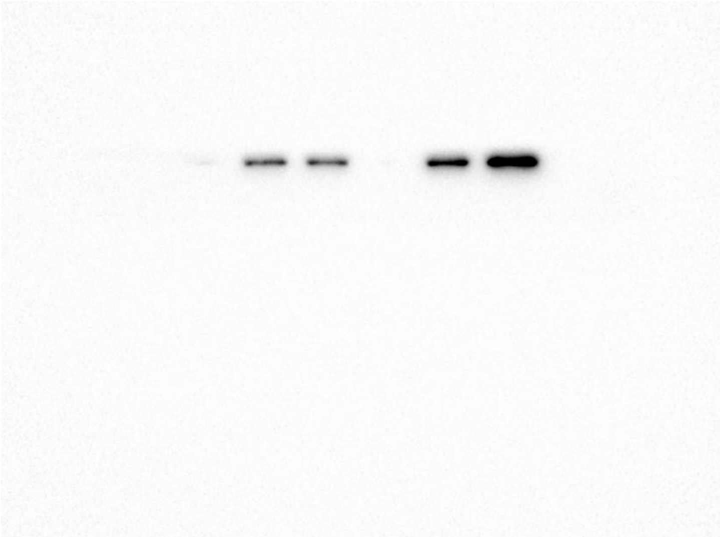

vitro_Ikb

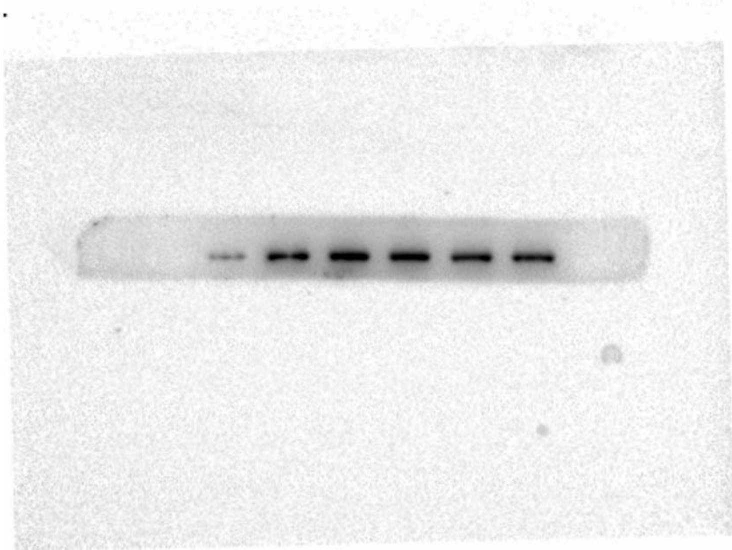
vitro_JNK

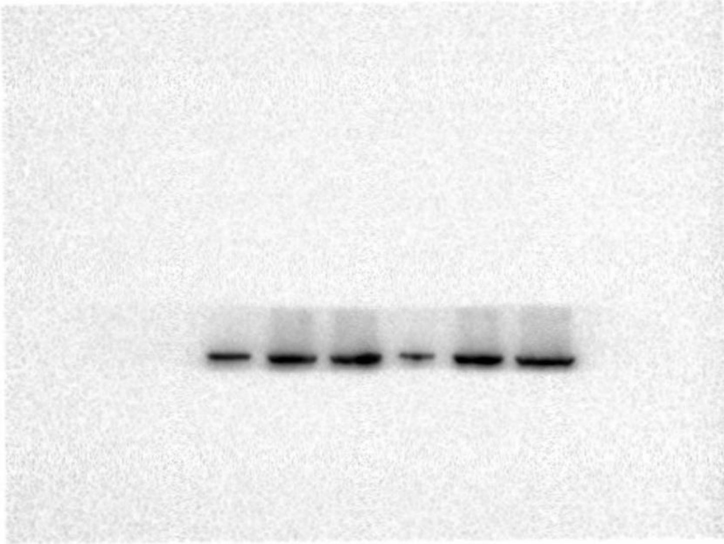
vitro_NFATc1

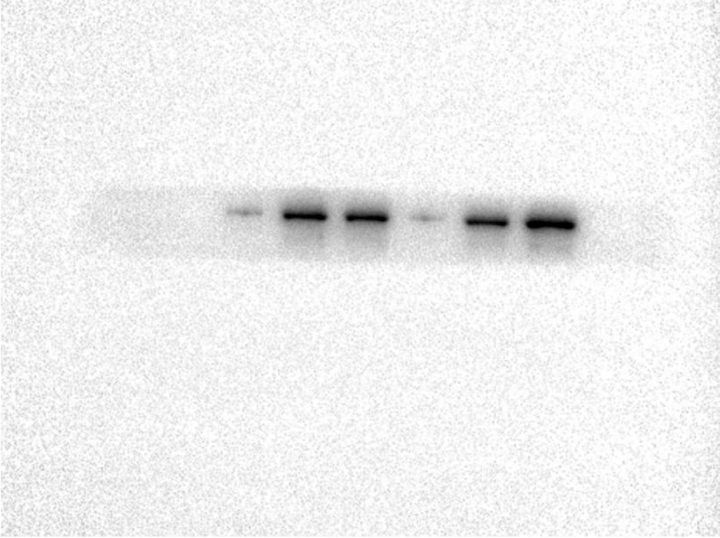
vitro_NFATc2

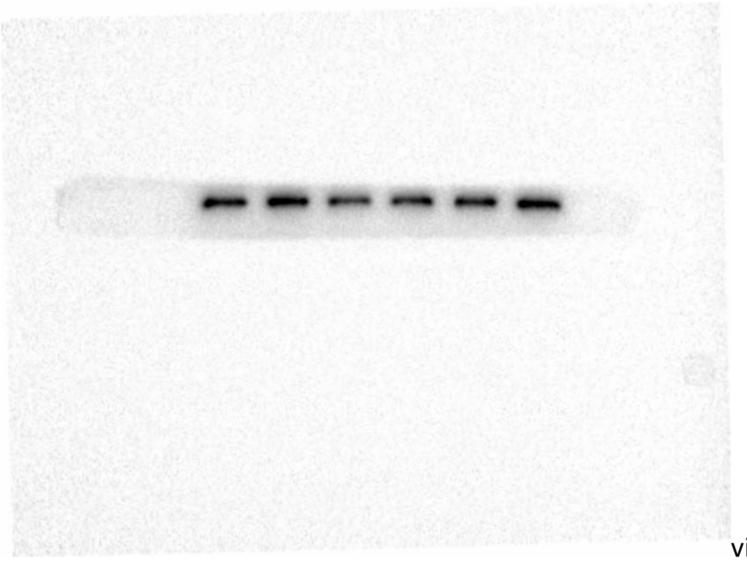

vitro_P38

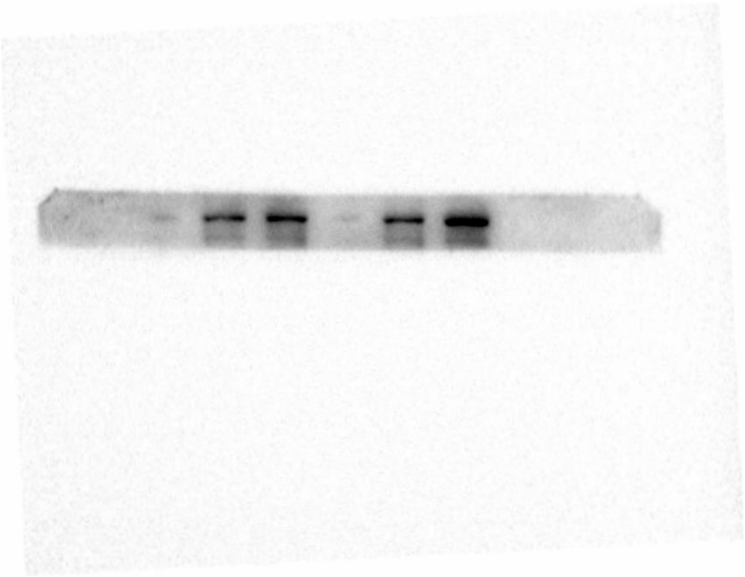

vitro_P65

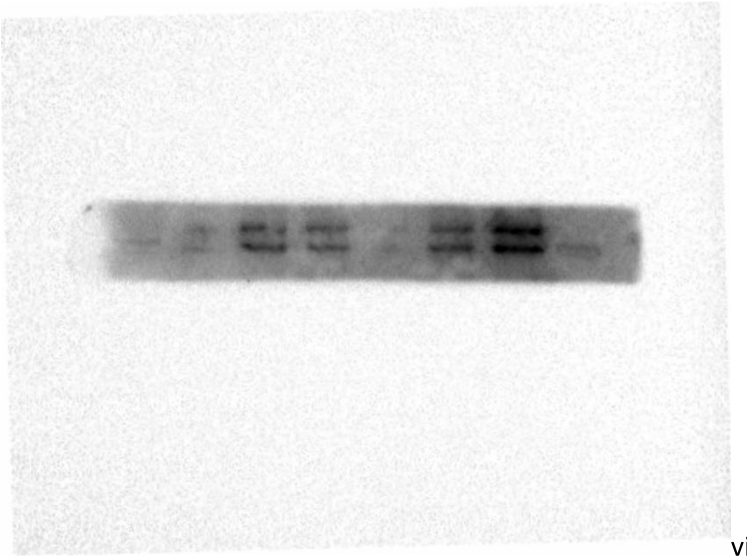

vitro_p-Erk

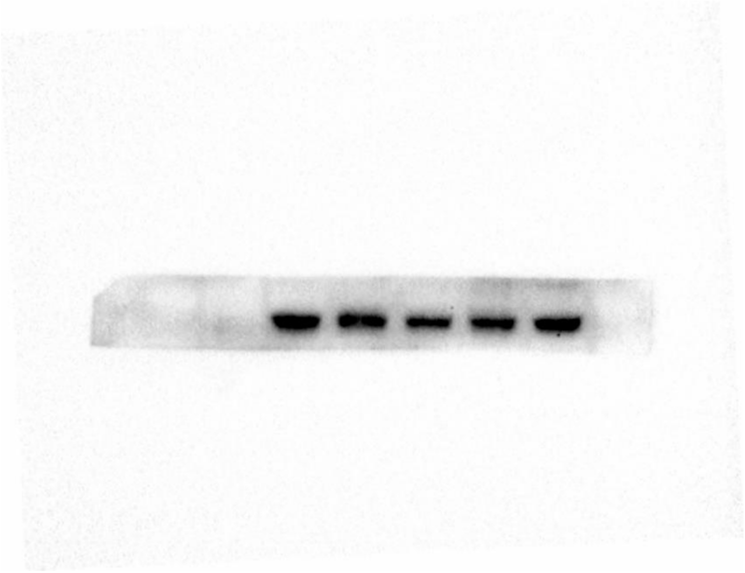

vitro_p-JNK

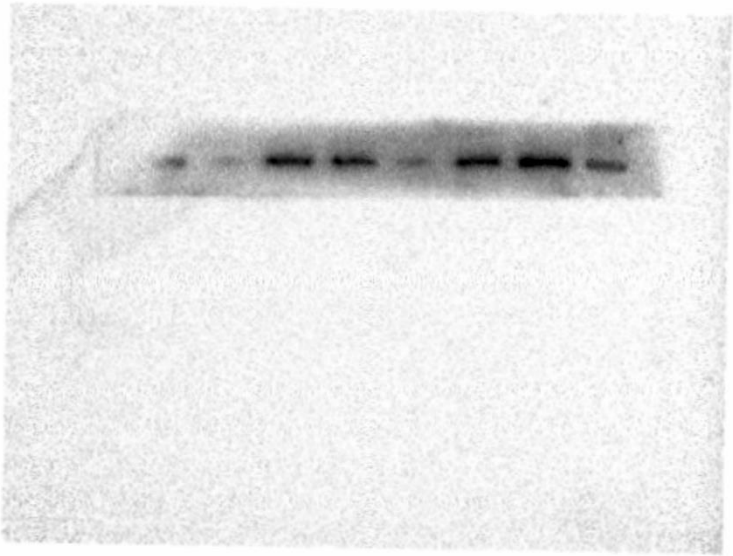

vitro_p-P38

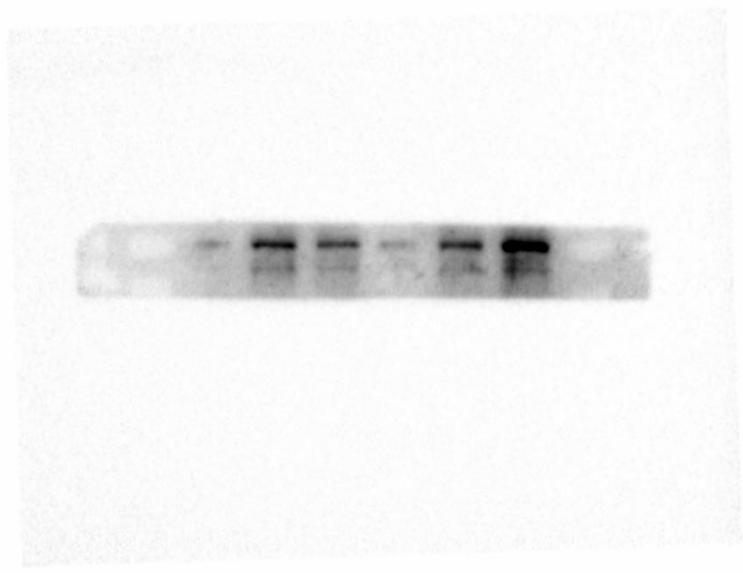

vitro_p-P65

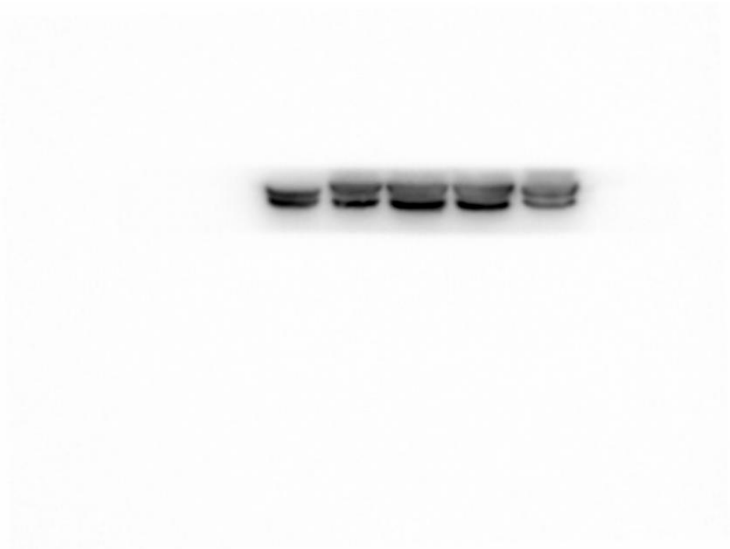

vivo_Erk

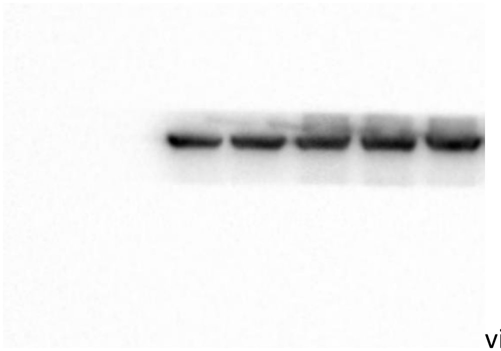

vivo_GAPDH

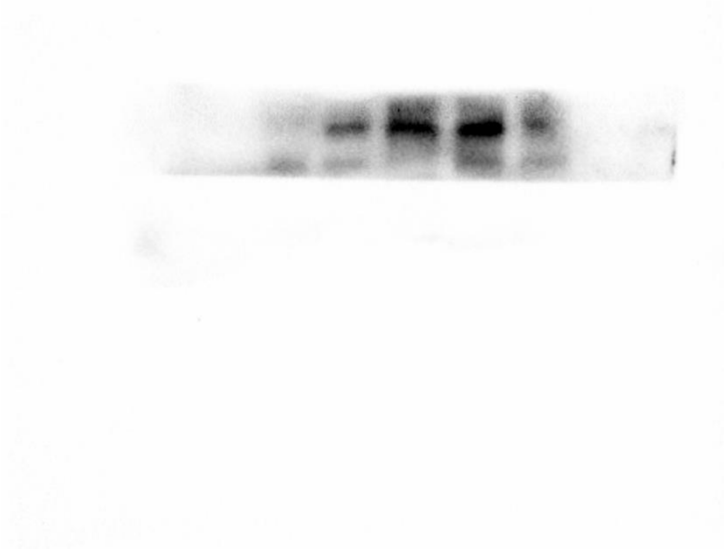
vivo_Ikb

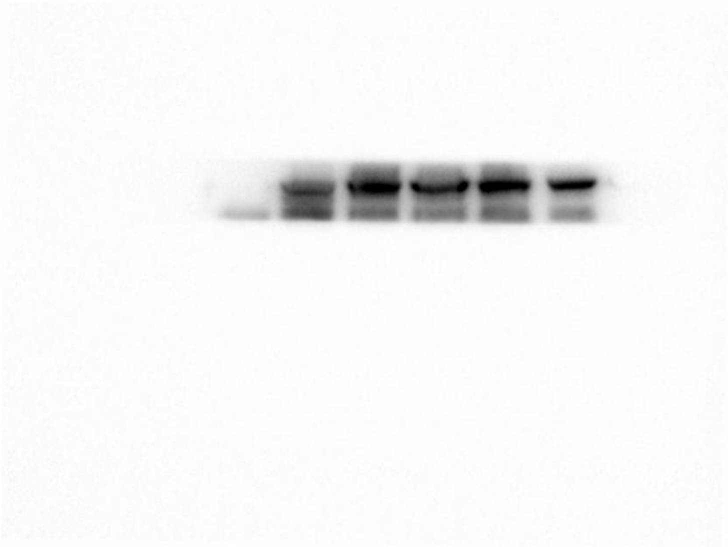
vivo_JNK

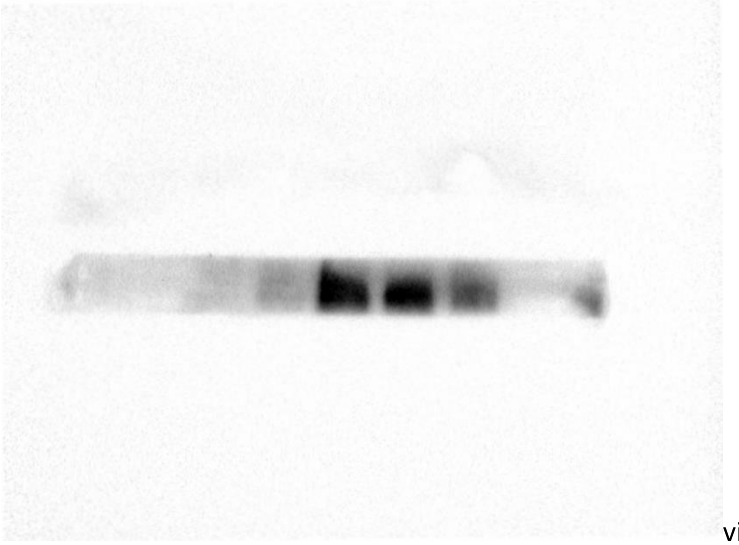
vivo_NFATc1

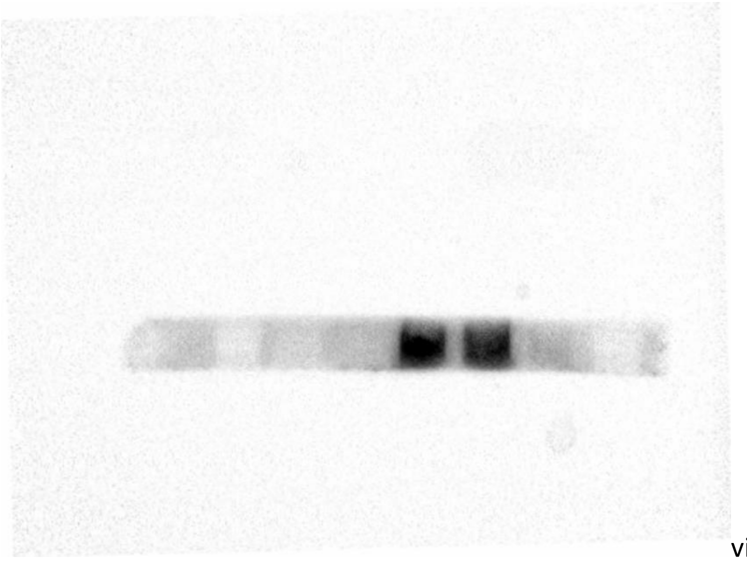

vivo_NFATc2

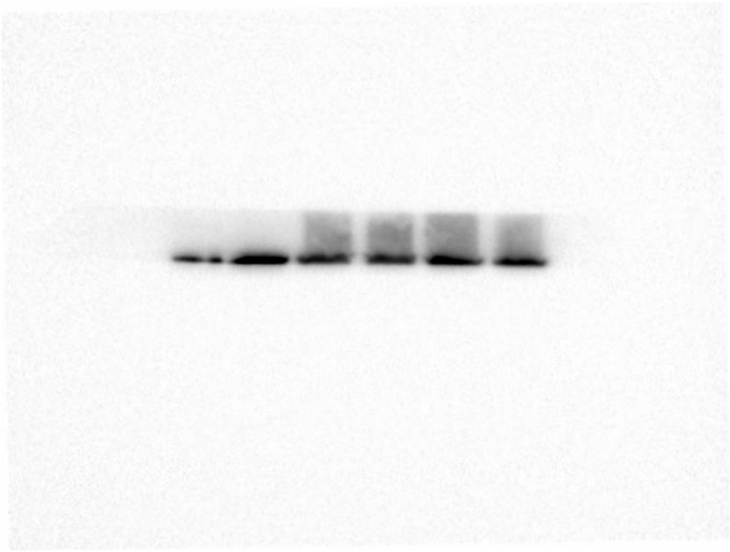

vivo_P38

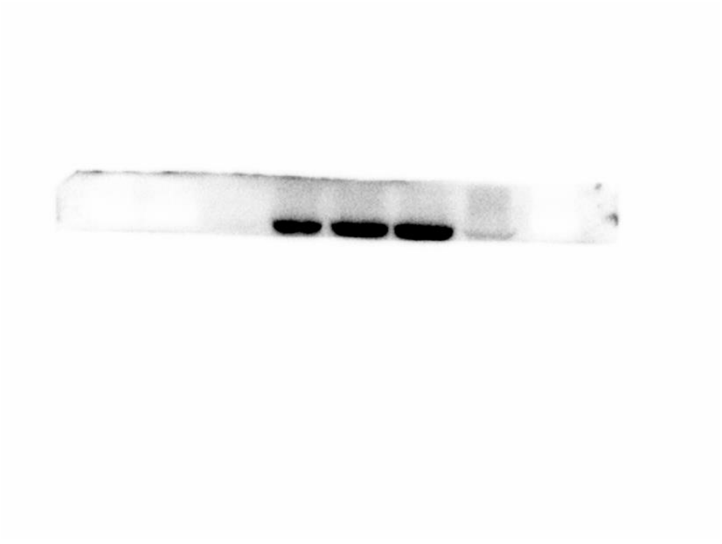

vivo_P65

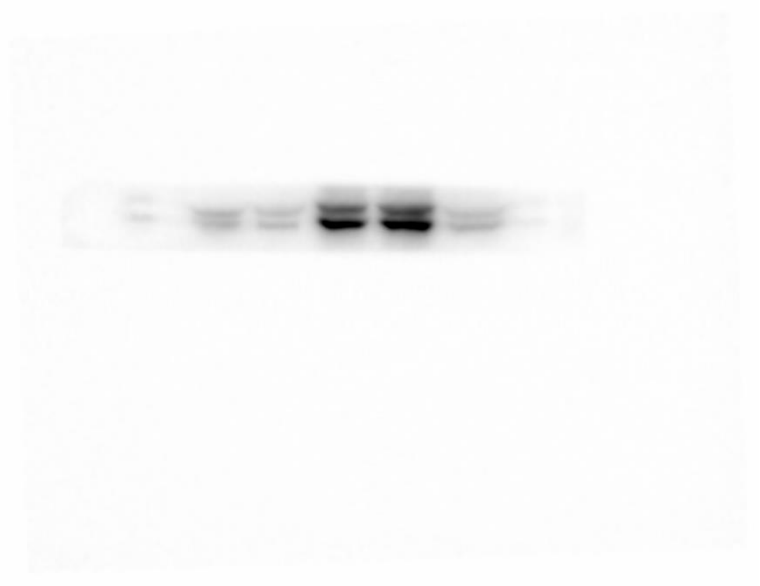

vivo_p-Erk

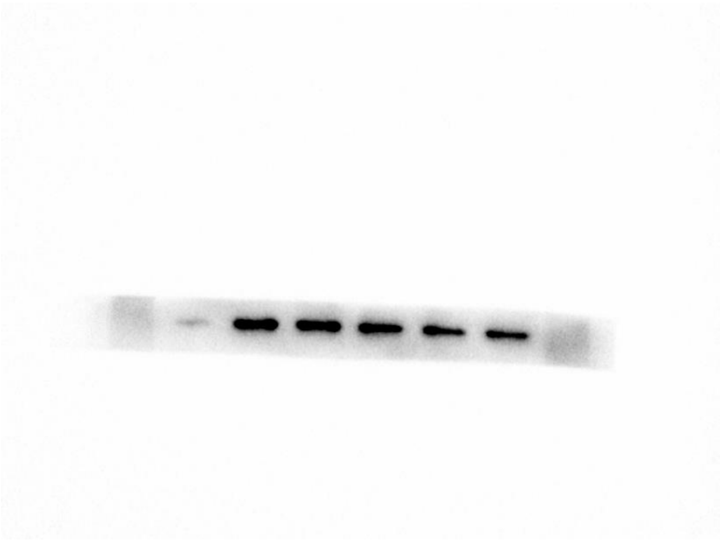

vivo_p-JNK

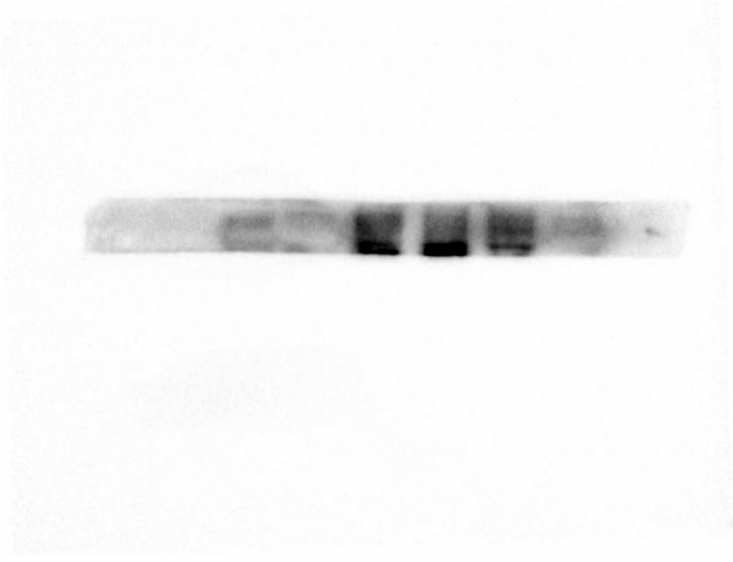

vivo_p-P38

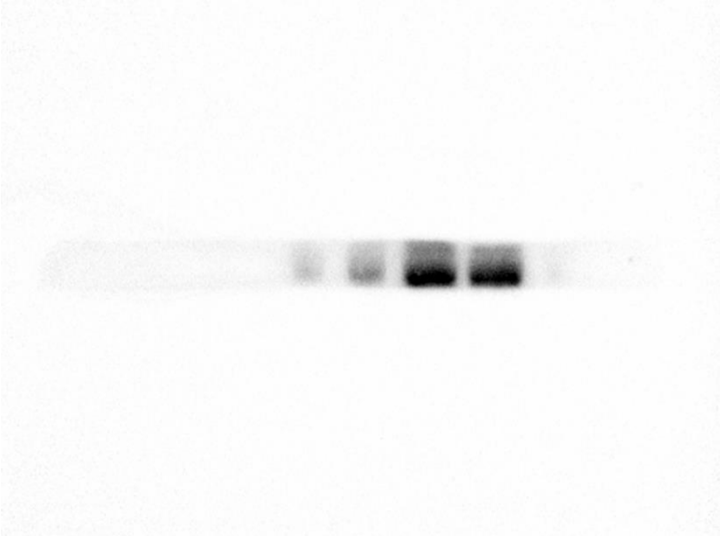

vivo_p-P65
